# Long noncoding RNA *LINC02582* acts downstream of miR-200c to promote radioresistance through CHK1 in breast cancer cells

**DOI:** 10.1038/s41419-019-1996-0

**Published:** 2019-10-10

**Authors:** Baiyao Wang, Jieling Zheng, Rong Li, Yunhong Tian, Jie Lin, Yingying Liang, Quanquan Sun, Anan Xu, Ronghui Zheng, Mengzhong Liu, Aimin Ji, Junguo Bu, Yawei Yuan

**Affiliations:** 1 Department of Radiation Oncology, Nanfang Hospital, Southern Medical University, Guangzhou, Guangdong Province People’s Republic of China; 2 Department of Radiation Oncology, Affiliated Cancer Hospital & Institute of Guangzhou Medical University, Guangzhou, Guangdong Province People’s Republic of China; 3 Department of Radiation Oncology, Zhejiang Cancer Hospital, Hangzhou, Zhejiang Province People’s Republic of China; 4 Department of Radiation Oncology, Zhujiang Hospital, Southern Medical University, Guangzhou, Guangdong Province People’s Republic of China

**Keywords:** Breast cancer, Radiotherapy

## Abstract

Radiotherapy is essential to treat breast cancer and microRNA (miRNA) miR-200c is considered as a radiosensitizer of breast cancer. However, the molecular mechanisms by which miR-200c regulates radiosensitivity remain largely unknown. In the present study, we showed that induction of miR-200c led to widespread alteration in long noncoding RNA (lncRNA) expression in breast cancer cells. We identified lncRNA *LINC02582* as a target of miR-200c. Inhibition of *LINC02582* expression increased radiosensitvity, while overexpression of *LINC02582* promoted radioresistance. Mechanistically, *LINC02582* interacts with deubiquitinating enzyme ubiquitin specific peptidase 7 (USP7) to deubiquitinate and stabilize checkpoint kinase 1 (CHK1), a critical effector kinase in DNA damage response, thus promoting radioresistance. Furthermore, we detected an inverse correlation between the expression of miR-200c vs. *LINC02582* and CHK1 in breast cancer samples. These findings identified *LINC02582* as a downstream target of miR-200c linking miR-200c to CHK1, in which miR-200c increases radiosensitivity by downregulation of CHK1.

## Introduction

Radiation therapy plays an important role in the multidisciplinary management of breast cancer. It not only provides local control and reduces tumor local relapse but also increases patients’ long-term survival and decreases their mortality^1,2^. However, radioresistance is a major cause of failure of breast cancer radiation therapy^3^. Therefore, the molecular mechanisms involved in breast cancer radioresistance should be investigated.

MicroRNAs (miRNAs) regulate a wide range of biological processes and dysregulation of miRNAs contributes to cancer^4,5^. MiR-200c is a member of the miR-200 superfamily and is involved in stemness, epithelial–mesenchymal transition, and chemoresistance in various cancer cells^6–8^. Moreover, miR-200c has been identified as a critical regulator of radiosensitivity in several types of cancer, including breast cancer. MiR-200c sensitizes breast cancer cells to radiation by targeting TBK1 and EGFR^9,10^. Our previous study also showed that miR-200c sensitizes breast cancer cells to radiation by inhibiting radiation-induced autophagy^11^. Importantly, therapeutic delivery of miR-200c markedly increased radiosensitivity in several types of cancer^12,13^. Thus, miR-200c acts as a tumor radiosensitizer and might represent an attractive target to increase the efficacy of radiation therapy for breast cancer. However, the molecular mechanisms by which miR-200c regulates radiosensitivity require further investigation.

Long noncoding RNAs (lncRNAs) are a large class of non-protein-coding transcripts with a length >200 bases^14^. LncRNAs have important roles in various biological processes in cancer, including proliferation, invasion, and metastasis^15,16^. For instance, lncRNA *HOTAIR* interacts with polycomb repressive complex 2 to reprogram chromatin, thus promoting breast cancer invasion and metastasis^17^. Furthermore, lncRNA *NKILA* is a negative regulator of NF-κB signaling, inhibiting NF-κB-mediated metastasis in breast cancer. Low *NKILA* expression predicts poor clinical outcome in patients with breast cancer^18^. In addition, lncRNA *EPIC1* is an oncogenic lncRNA that interacts with MYC to promote cell-cycle progression in breast cancer. High expression of *EPIC1* is associated with poor prognosis in patients with breast cancer^19^. Therefore, lncRNAs represent a wide range of potential targets for cancer treatment. However, the role of lncRNAs in radiosensitivity is unclear.

Studies have shown that miRNAs interact with lncRNAs to regulate lncRNA levels^20^. For example, miR-211 inhibits lncRNA *loc285194* expression^21^; miR-17-3p directly targets lncRNA *GCASPC* and decreases its half-life^22^; and miR-193b suppresses lncRNA *MIR31HG* expression^23,24^. Given that miR-200c can increase the radiosensitivity of breast cancer cells and that the contribution of miR-200c to lncRNA expression has not been assessed, we hypothesized that lncRNAs might be critical downstream targets of miR-200c in regulating radiosensitivity.

In the present study, we used microarray analysis to delineate the alterations in lncRNA expression induced by miR-200c. We identified lncRNA *LINC02582* as a downstream target of miR-200c. *LINC02582* is required for radioresistance in breast cancer cells. Mechanistically, *LINC02582* interacts with deubiquitinating enzyme ubiquitin specific peptidase 7 (USP7) to deubiquitinate and stabilize checkpoint kinase 1 (CHK1), thereby promoting radioresistance.

## Results

### Overexpression of miR-200c enhances the radiosensitivity of breast cancer cells

**Fig. 1:**
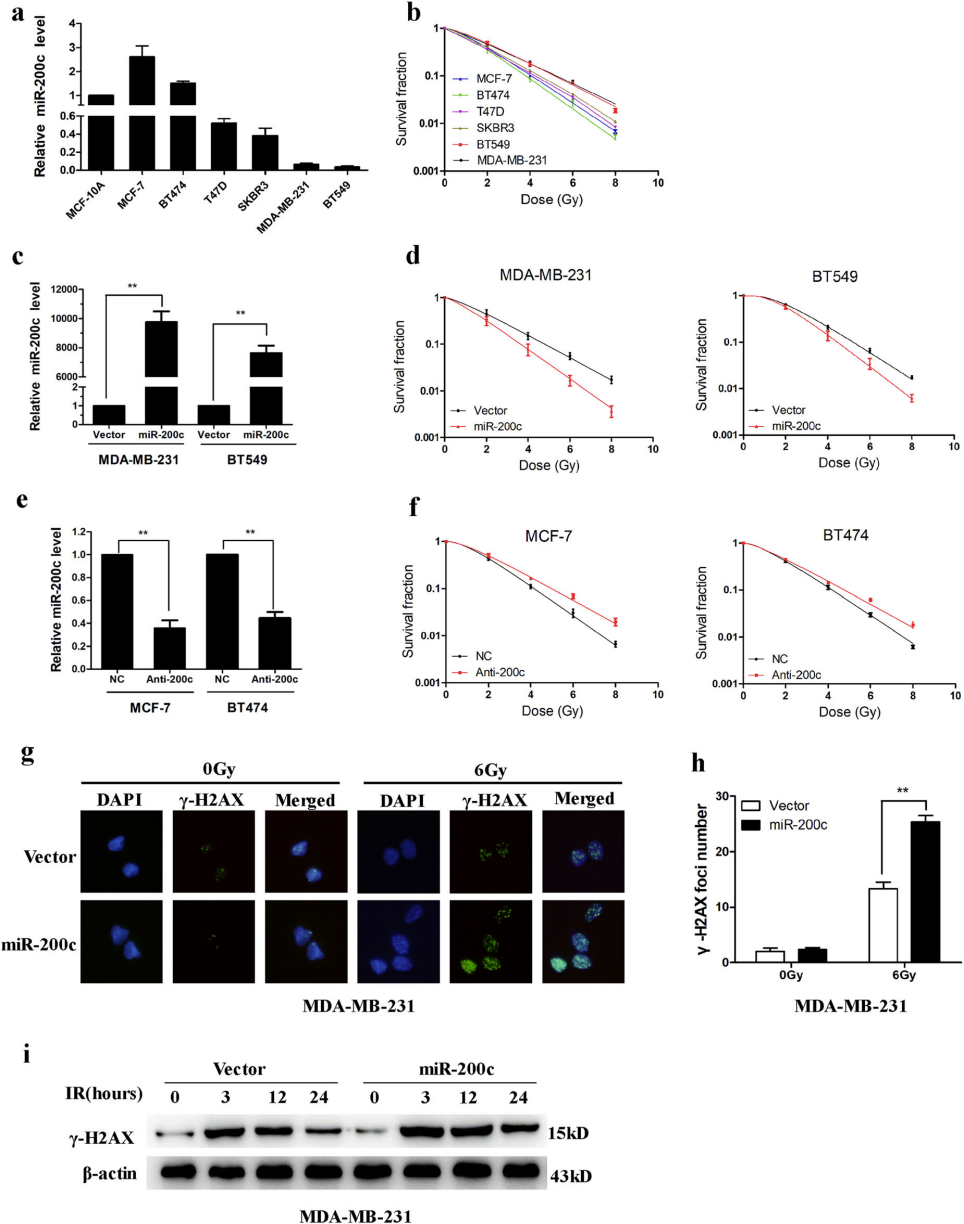
MiR-200c overexpression increases the radiosensitivity of breast cancer cells. **a** Relative expression of miR-200c in breast cancer cells and MCF-10A cells were detected using qRT-PCR. **b** Clonogenic survival assays of MDA-MB-231, BT549, SKBR3, T47D, BT474, and MCF-7 cells. **c** Relative expression of miR-200c in MDA-MB-231 and BT549 cells transduced with lentivirus encoding miR-200c or the empty vector. **d** Clonogenic survival assays of MDA-MB-231 and BT549 cells transduced with miR-200c. **e** Relative expression of miR-200c in MCF-7 and BT474 cells after transfection the miR-200c inhibitor. **f** Clonogenic survival assays of MCF-7 and BT474 cells transfected with the miR-200c inhibitor. **g**, **h** γ-H2AX foci formation analyzed by immunofluorescence in MDA-MB-231 cells transduced with miR-200c or empty vector, 24 h after 6 Gy IR. **i** Western blotting analysis of γ-H2AX expression in MDA-MB-231 cells transduced with miR-200c, at the indicated time points after 6 Gy IR. Data are presented as means ± SD, *n* = 3, *P < 0.05, **P < 0.01

To confirm that miR-200c sensitizes breast cancer cells to radiation, we first determined the miR-200c expression level in several breast cancer cell lines. Consistent with a previous report^25^, miR-200c is commonly expressed in breast cancer cells (Fig. 1a). Compared with miR-200c high-expression cell lines (MCF-7, BT474), miR-200c low-expression cell lines (MDA-MB-231, BT549, SKBR3, T47D) showed higher clonogenic survival after irradiation (Fig. 1b). These data indicated a positive correlation between miR-200c expression and radiosensitivity. MDA-MB-231 and BT549 cells were transduced with lentivirus expressing miR-200c (Fig. 1c). Overexpression of miR-200c reduced the survival fraction of MDA-MB-231 and BT549 cells subjected to irradiation (Fig. 1d). Conversely, inhibition of miR-200c increased the survival fraction of MCF-7 and BT474 cells after irradiation (Fig. 1e, f). Irradiation caused double-stranded DNA breaks (DSBs) with formation of γ-H2AX foci, which indicated delayed repair and correlated with radiosensitivity. Indeed, miR-200c overexpression led to persistence of γ-H2AX foci in MDA-MB-231 cells at 24 h after irradiation (Fig. 1g, h). Analysis of γ-H2AX protein levels showed that miR-200c overexpression significantly increased γ-H2AX levels after irradiation (Fig. 1i). These results confirmed that overexpression of miR-200c suppresses DNA repair and sensitizes breast cancer cells to radiation.

### Identification of miR-200c-associated lncRNAs

**Fig. 2:**
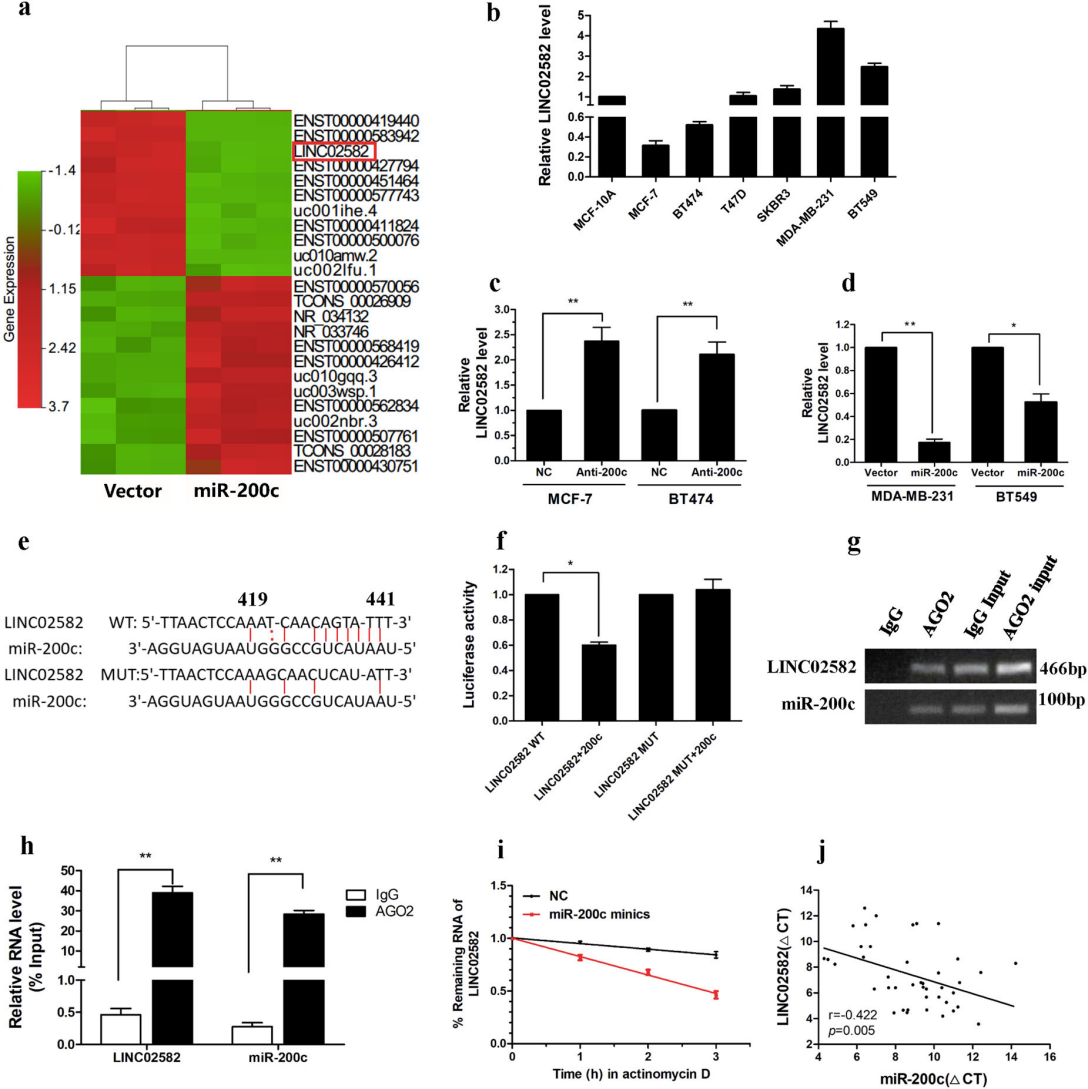
*LINC02582* is a target of miR-200c. **a** Representative heat map of the lncRNAs that were most differentially expressed between miR-200c-overexpressing cells and control cells. **b** Relative expression of *LINC02582* in breast cancer cells and MCF-10A cells. **c** Relative expression of *LINC02582* in MCF-7 and BT474 cells after transfection with the miR-200c inhibitor. **d** Relative expression of *LINC02582* in MDA-MB-231 and BT549 cells after transduction with miR-200c. **e** Target site of miR-200c in the *LINC02582* sequence, as predicted by TargetScan and miRBase (upper panel). **f** Relative luciferase activity of MDA-MB-231 cells after co-transfection with wild type or mutant *LINC02582* vectors and an miR-200c mimic. **g** Anti-AGO2 RIP to investigated AGO2 binding to miR-200c and *LINC02582*; IgG was used as a negative control. **h** The levels of miR-200c and *LINC02582* were detected by qRT-PCR and presented as fold enrichment of AGO2 relative to input. **i** The half-life of *LINC02582* was shortened by miR-200c overexpression. MDA-MB-231 cells were transfected with miR-200c mimics, after 48 h, a time course for RNA stability was started by adding the actinomycin D. The cells were harvested at the indicated time points. The expression levels of *LINC02582* were detected by qRT-PCR. **j** Expression levels of *LINC02582* and miR-200c were negatively correlated in 42 breast cancer samples, as measured by qRT-PCR. The ΔCT values were subjected to Pearson correlation analysis. Data are presented as means ± SD, *n* = 3, *P *<* 0.05, **P *<* 0.01

To identify miR-200c-associated lncRNAs, differential expression of lncRNAs between MDA-MB-231 cells stably overexpressing miR-200c and control cells was assessed using microarray analysis. Hierarchical clustering showed variations in lncRNA expression between miR-200c-overexpressing cells and control cells (Fig. 2a). We found that 424 lncRNAs were upregulated and 521 lncRNAs were downregulated (fold change > 2, *P* *<* 0.05) in miR-200c-overexpressing cells. To validate the results of microarray analysis, we randomly selected 10 lncRNAs showing >10-fold change and determined their expression using qRT-PCR. Our data were consistent with the microarray results (Supplementary Fig. 1a). Previous studies demonstrated that interactions between miRNAs and lncRNAs represent an important gene regulation pathway^20^. Among the downregulated lncRNAs, we predicted 58 potential lncRNA targets for miR-200c using the TargetScan and miRbase prediction algorithm (Supplementary Table 1). Taken together, the results suggested that overexpression of miR-200c lead to widespread alterations in lncRNA expression.

### *LINC02582* is a direct target of miR-200c

To investigate lncRNAs regulated by miR-200c, we selected five most differentially expressed candidate lncRNAs from among the potential targets of miR-200c and determined their expression in breast cancer cells (Supplementary Fig. 1b). Interestingly, among the five candidate lncRNAs, we found that *LINC02582* (NCBI number *NR_038340*) expression was higher in the radioresistant breast cancer lines, including MDA-MB-231, BT549, SKBR3, T47D with low miR-200c levels, than in the radiosensitive cell lines, including MCF-7, BT474 with high miR-200c levels (Fig. 2b). This result revealed a negative correlation between *LINC02582* expression and miR-200c expression and implied that *LINC02582* was a possible target of miR-200c. Therefore, *LINC02582* was chosen for further investigation. *LINC02582* is located on human chromosome 18q22.3. The sequence of *LINC02582* was identified by 5′ and 3′ RACE. Analysis of *LINC02582* sequences by Open Reading Frame Finder from NCBI failed to predict an encoded protein, suggesting that *LINC02582* has no protein-coding potential (Supplementary Table 2).

To investigate whether miR-200c regulates the expression of *LINC02582*, we suppressed miR-200c expression in MCF-7 and BT474 cells, which led to a significant increase in *LINC02582* expression (Fig. 2c). Conversely, ectopic expression of miR-200c resulted in a significant reduction in *LINC02582* in MDA-MB-231 and BT549 cells (Fig. 2d). However, we found no significant difference in miR-200c expression between *LINC02582* knockdown or overexpression cells (Supplementary Fig. 2a). Subsequently, TargetScan and miRBase predicted the existence of interactions between miR-200c and *LINC02582* (Fig. 2e). To confirm the binding between *LINC02582* and miR-200c, we performed dual-luciferase assays, which showed that miR-200c decreased the luciferase activity of the wild-type *LINC02582* vector, but not that of the mutant *LINC02582* vector (Fig. 2f). MiRNAs are known to bind their targets and cause translational repression or RNA degradation in an AGO2-dependent manner. To test whether miR-200c regulates *LINC02582* in such a manner, we conducted an RNA immunoprecipitation (RIP) experiment in MDA-MB-231 cells using anti-AGO2 antibodies. The results showed that *LINC02582* and miR-200c were enriched in AGO2 immunoprecipitates relative to IgG immunoprecipitates, which confirmed direct binding between miR-200c and *LINC02582* (Fig. 2g, h). Moreover, ectopic expression of miR-200c shortened the half-life of *LINC02582* (Fig. 2i). Finally, qRT-PCR revealed a negative correlation between miR-200c and *LINC02582* expression in 42-paired samples of breast cancer tissue (Fig. 2j). These results indicated that *LINC02582* is a target of miR-200c.

### *LINC02582* inhibition increases radiosensitivity of breast cancer cells

**Fig. 3:**
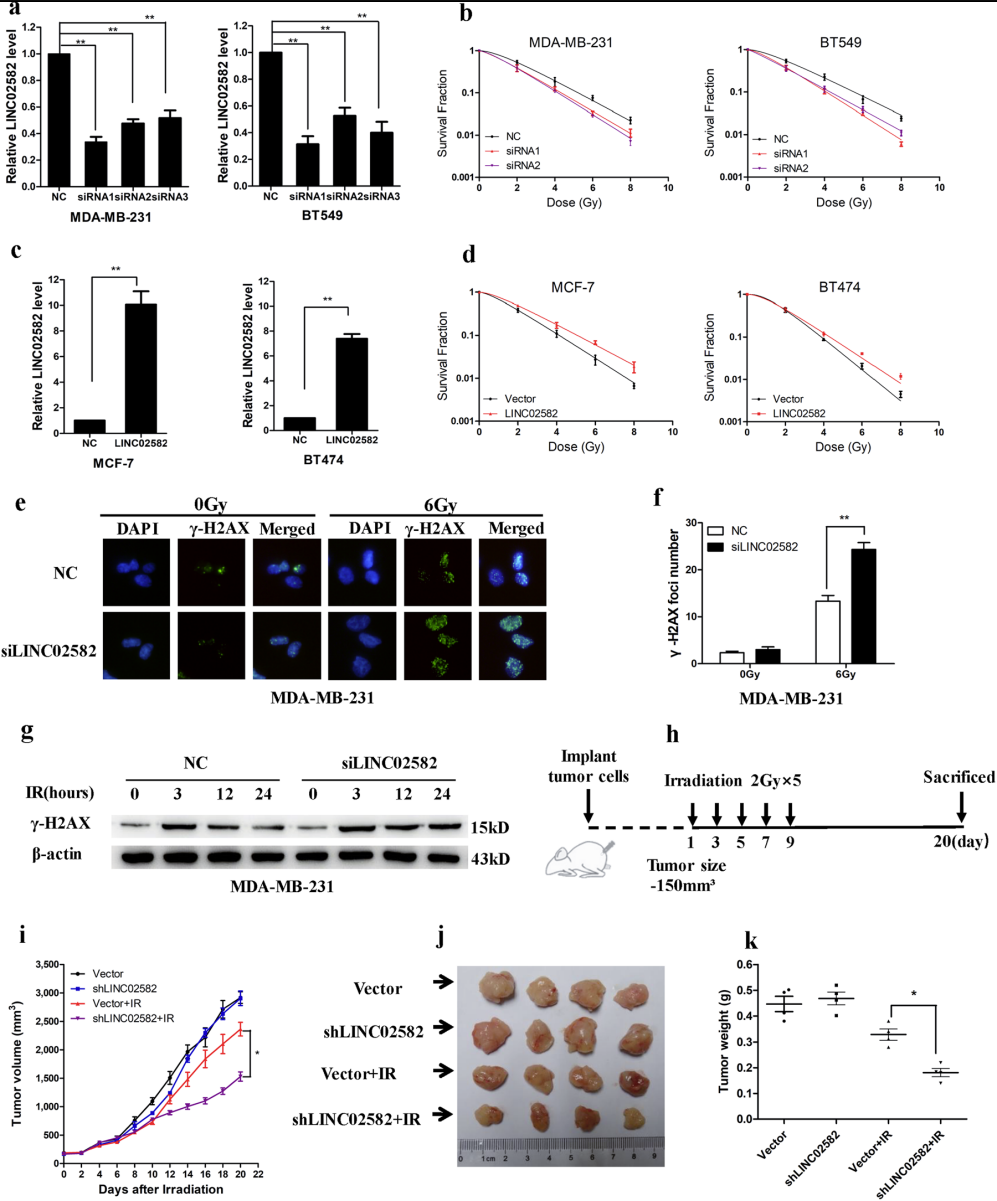
*LINC02582* promotes radioresistance of breast cancer cells. **a** Expression of *LINC02582* in MDA-MB-231 and BT549 cells transfected with three different siRNAs corresponding to *LINC02582*. **d** Clonogenic survival assays of MDA-MB-231 and BT549 cells transfected with two different *LINC02582* siRNAs. **c** Expression of *LINC02582* in MCF-7 and BT474 cells transduced with lentivirus encoding the *LINC02582* sequence. **d** Clonogenic survival assay of MCF-7 and BT474 cells transduced with *LINC02582*. **e**, **f** Formation of γ-H2AX foci at 24 h after 6 Gy IR, analyzed by immunofluorescence in MDA-MB-231 cells transfected with *LINC02582* siRNA1. Data are presented as means ± SD, *n* = 3, *P *<* 0.05, **P *<* 0.01. **g** Western blotting analysis of γ-H2AX expression in MDA-MB-231 cells transfected with *LINC02582* siRNA1, at the indicated time points after 6 Gy IR. **h** Schemes for the establishment and treatment of the breast cancer orthotopic mouse model. **i** Tumor size in mice bearing control MDA-MB-231 xenografts or *LINC02582* shRNA-transduced MDA-MB-231 xenografts. Data points show the mean tumor volume (mm^3^) of each group (*n* = 4); bars, SE. *P *<* 0.05. **j** Photographs of tumors developing in normal control (Vector), sh*LINC02582*, Vector + irradiation (Vector + IR), and sh*LINC02582* + IR mice are presented. **k** Weight of tumors from the mouse model; tumor weights in the sh*LINC02582* + IR mice were lower than those from the Vector + IR mice. Data points show the mean tumor weight (**g**) of each group (*n* = 4); bars, SE. *P *<* 0.05

To evaluate the biological functions of *LINC02582*, we inhibited its expression in MDA-MB-231 and BT549 cells (Fig. 3a), which had no effect on the viability of the breast cancer cells (Supplementary Fig. 2b). Interestingly, inhibition of *LINC02582* expression reduced the surviving fraction of MDA-MB-231 and BT549 cells after irradiation (Fig. 3b). Conversely, overexpression of *LINC02582* increased the surviving fraction of MCF-7 and BT474 cells after irradiation (Fig. 3c, d). Furthermore, *LINC02582* silencing led to persistence of γ-H2AX foci in MDA-MB-231 cells and BT549 cells at 24 h after irradiation (Fig. 3e, f, Supplementary Fig. 2c, d). *LINC02582* silencing also markedly increased γ-H2AX expression after irradiation. (Fig. 3g, Supplementary Fig. 2e). These results indicated that silencing of *LINC02582* enhanced the radiosensitivity of breast cancer cells. To further investigate the effects of *LINC02582* in vivo, MDA-MB-231 cells with *LINC02582* knockdown were used to create a xenograft model (Supplementary Fig. 2f). When the tumor volume reached 150 mm^3^, local tumor irradiation was performed using a 2-Gy fractionated dose every other day for 10 days (Fig. 3i). Knockdown of *LINC02582* had no effect on the growth of non-irradiated tumors, but caused tumor growth inhibition after irradiation (Fig. 3j, k). Taken together, these results demonstrated that silencing of *LINC02582* enhanced the radiosensitivity of breast cancer cells both in vitro and in vivo.

### *LINC02582* interacts with USP7

**Fig. 4:**
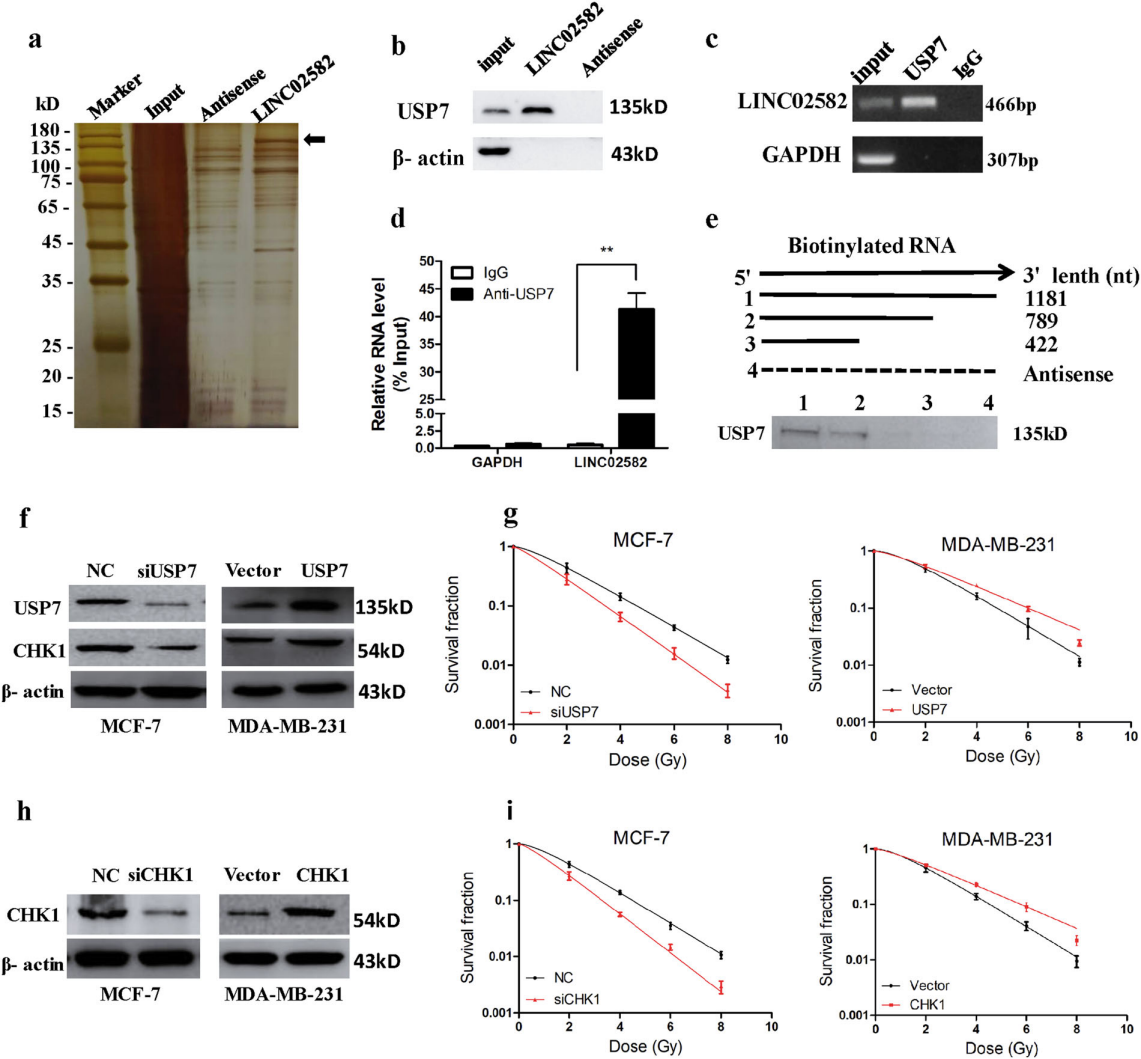
*LINC02582* interacts with USP7. **a** Highlighted regions (arrows) were subjected to mass spectrometry for identification and USP7 was identified as the band unique to *LINC02582*. **b** Western blotting analysis showing the specific interaction of *LINC02582* with USP7. **c** RIP was performed using anti-USP7 antibodies and specific primers to detect *LINC02582* or GAPDH. **d** Enrichment of RNA immunoprecipitation was determined as the amount of RNA associated with immunoprecipitation of USP7 relative to the input control. **e** RNAs corresponding to different fragments of *LINC02582* or its antisense sequence (dotted line) were biotinylated and incubated with MDA-MB-231 whole-cell extracts, targeted with streptavidin beads, and washed. The interaction with USP7 was then detected using western blotting. **f** Western blotting analysis of USP7 and CHK1 protein levels in breast cancer cells. MCF-7 cells were transfected with USP7 siRNA and MDA-MB-231 cells were transfected with the USP7 expression plasmid. **g** The survival fraction of MCF-7 and MDA-MB-231 cells. MCF-7 and MDA-MB-231 cells treated as in **f** and then exposed to irradiation. **h** Western blotting analysis of CHK1 protein levels; MCF-7 cells were transfected with CHK1 siRNA and MDA-MB-231 cells were transfected with the CHK1 expression plasmid. **i** The survival fraction of MCF-7 cells and MDA-MB-231 cells. MCF-7 and MDA-MB-231 cells treated as in **h** and then exposed to irradiation. Data are presented as means ± SD, *n* = 3, *P *<* 0.05, **P *<* 0.01

Studies have shown that some lncRNAs perform their functions by interacting with specific proteins^26,27^. To evaluate whether *LINC02582* acts via this mechanism, we performed RNA pull-down assays to identify proteins that interact with *LINC02582*, followed by mass spectrometry analysis of the specific protein band for *LINC02582* (Supplementary Table 3). Among the proteins identified by mass spectrometry, USP7 was also detected by western blotting (Fig. 4a, b). An RIP experiment using anti-USP7 antibodies in extracts from MDA-MB-231 cells showed enrichment of *LINC02582* (but not *GAPDH* mRNA) using the USP7 antibodies vs. a nonspecific IgG control antibody (Fig. 4c, d). Moreover, deletion mapping analysis identified a 422–789 nt region of *LINC02582* that was required for the interaction between *LINC02582* and USP7 (Fig. 4e). Taken together, these results suggested that a specific interaction occurs between USP7 and *LINC02582*.

### *LINC02582* functions through its interaction with USP7

USP7 is a deubiquitinating enzyme involved in regulating the stability of many proteins^28–30^. USP7 promotes radioresistance of breast cancer cells by controlling CHK1 protein stability via direct deubiquitination^31^. Consistently, inhibition of USP7 reduced CHK1 protein levels and decreased radiosensitivity, while overexpression of USP7 elevated CHK1 protein levels and promoted radioresistance (Fig. 4f, g, Supplementary Fig. 3a). CHK1 is a critical effector kinase in the DNA damage response, which facilitates DNA damage repair and promotes radioresistance of breast cancer cells^32^. As expected, knockdown of *CHK1* sensitized MCF-7 cells to radiation, whereas overexpression of CHK1 resulted in radioresistance of MDA-MB-231 cells (Fig. 4h, i, Supplementary Fig. 3b). We hypothesized that the interaction between *LINC02582* and USP7 would lead to deubiquitination and stabilization of CHK1, which in turn would promote radioresistance.

**Fig. 5:**
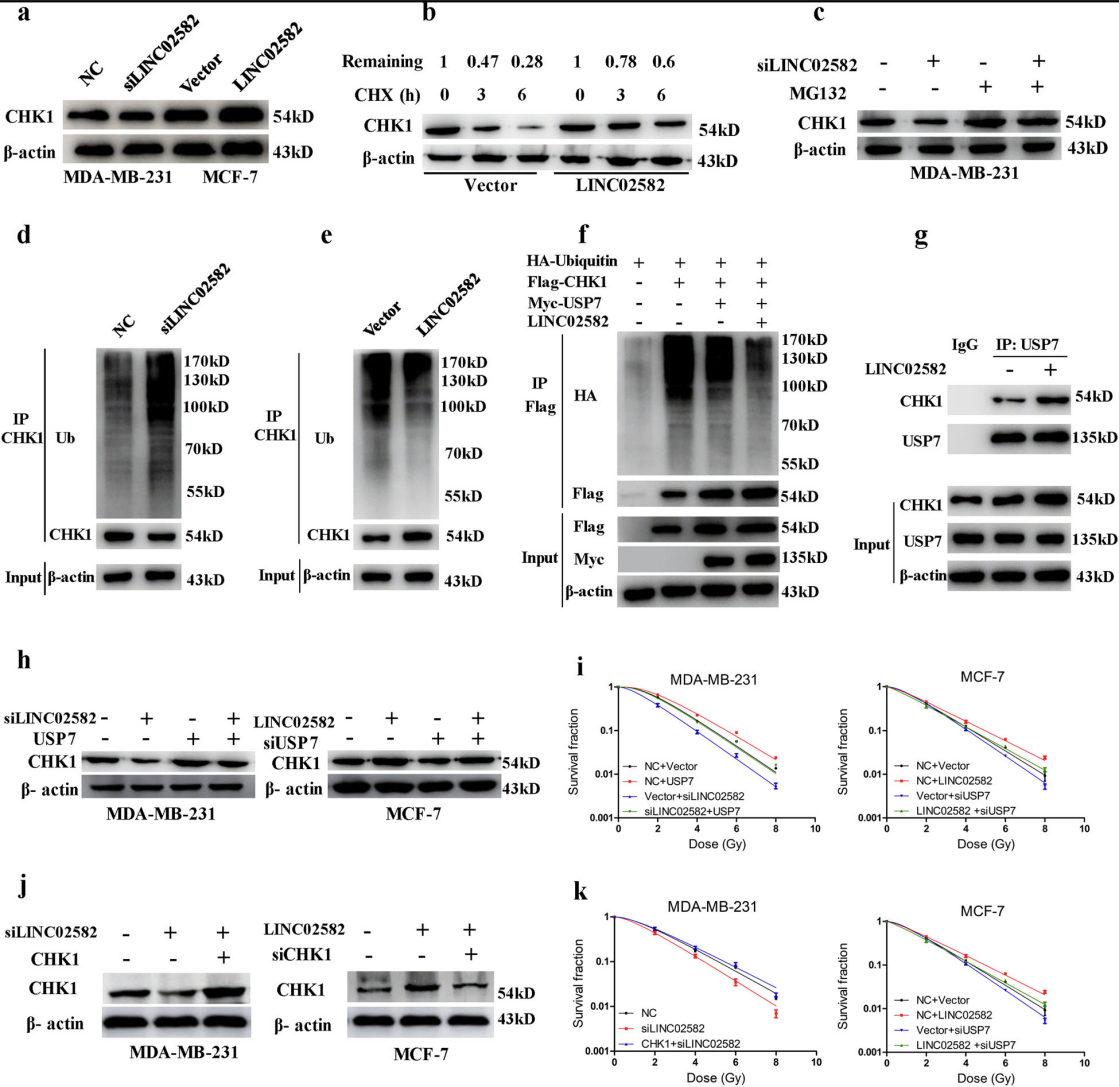
*LINC02582* functions through its interaction with USP7. **a** The CHK1 protein level was analyzed by western blotting. MDA-MB-231 cells were transfected with *LINC02582* siRNA1 and MCF-7 cells were transduced with *LINC02582*. **b** MCF-7 cells expressing *LINC02582* or empty vector were treated with CHX (20 μg/ml) for the indicated times. Whole-cell extracts were prepared and analyzed by western blotting. **c** MDA-MB-231 cells transfected with *LINC02582* siRNA1 were treated with MG132 (10 µM) for 24 h. Cell lysates were then analyzed by western blotting. **d** MDA-MB-231 cells were transfected with *LINC02582* siRNA1 and treated with MG132. Lysates were immunoprecipitated with anti-CHK1 antibody and the immunoprecipitates and input were analyzed by western blotting with the indicated antibodies. **e** MCF-7 cells were transduced with *LINC02582* and treated with MG132. Lysates were immunoprecipitated with anti-CHK1 antibody and the immunoprecipitates and input were analyzed by western blotting with the indicated antibodies. **f** The 293 T cells were co-transfected with the indicated expression plasmids and treated with MG132. Lysates were immunoprecipitated with anti-Flag antibody and the immunoprecipitates and input were analyzed by western blotting with the indicated antibodies. **g** MCF-7 cells were transduced with *LINC02582*. Lysates were immunoprecipitated with anti-USP7 antibody and the immunoprecipitates and input were analyzed by western blotting with the indicated antibodies. **h** Western blotting analysis of CHK1 protein expression. MDA-MB-231 cells co-transfected with the USP7 expression plasmid and *LINC02582* siRNA1. MCF-7 cells co-transfected with USP7 siRNA and *LINC02582*. **i** The survival fraction of MDA-MB-231 and MCF-7 cells treated as indicated. **j** Western blotting analysis of CHK1 protein expression. MDA-MB-231 cells were transfected with *LINC02582* siRNA1 alone or in combination with the CHK1 expression plasmid, while MCF-7 cells were transduced with *LINC02582* alone or in combination with CHK1 siRNA. **k** Clonogenic survival assays of MDA-MB-231 and MCF-7 cells. MDA-MB-231 and MCF-7 cells were treated as indicated

To test this hypothesis, we first showed that knockdown of *LINC02582* reduced the CHK1 level, while ectopic expression of *LINC02582* increased the CHK1 level (Fig. 5a). Importantly, *LINC02582* inhibition or ectopic expression did not affect the *CHK1* mRNA level (Supplementary Fig. 3c, d). To assess the effect of *LINCO2582* on CHK1 stability, we treated MCF-7 cells with cycloheximide (CHX) to inhibit protein synthesis and detected the level of remaining CHK1 using western blotting. The abundance of CHK1 in *LINC02582*-overexpressing cells remained relatively higher than that in the control cells (Fig. 5b), suggesting that *LINC02582* overexpression stabilized CHK1. Moreover, treatment of MDA-MB-231 cells silenced for *LINC02582* with the proteasome inhibitor MG132 resulted in increased endogenous CHK1 protein levels compared with those in control cells (Fig. 5c), suggesting that the ubiquitin–proteasome pathway plays a critical role in the *LINC02582*-mediated upregulation of CHK1.

Indeed, inhibition of *LINC02582* in MDA-MB-231 cells promoted endogenous ubiquitination of CHK1 (Fig. 5d). On the contrary, overexpression of *LINC02582* in MCF-7 cells induced endogenous deubiquitination of CHK1 (Fig. 5e); however, inhibition of USP7 blocked the deubiquitination inducted by *LINC02582* overexpression (Supplementary Fig. 4a). Furthermore, to detect the ubiquitination of CHK1, the 293 T cells were co-transfected with HA-tagged Ubiquitin, Flag-tagged CHK1, Myc-tagged USP7, and *LINC02582* expression plasmids. Consistently, overexpression of USP7 significantly induced deubiquitination of CHK1 in 293 T cells; importantly, overexpression of *LINC02582* promoted USP7-induced deubiquitination of CHK1 (Fig. 5f). Finally, we found that overexpression of *LINC02582* could enhance the interaction between USP7 and CHK1 (Fig. 5g). Collectively, these results suggested that *LINC02582* enhances the interaction between USP7 and CHK1, thereby increasing the deubiquitination of CHK1.

Importantly, we observed that the reduction in CHK1 caused by inhibition of *LINC02582* was reversed by USP7 overexpression, while the upregulation of CHK1 caused by *LINC02582* ectopic expression could be reversed by USP7 knockdown (Fig. 5h). The induction of radiosensitivity by *LINC02582* inhibition was blocked by USP7 overexpression; however, the radioresistance induced by ectopic expression of *LINC02582* could be blocked by USP7 knockdown (Fig. 5i). Similarly, ectopic expression of CHK1 in *LINC02582*-knockdown cells rescued the radioresistance, whereas knockdown of *CHK1* reversed the *LINC02582*-induced radioresistance (Fig. 5j, k). These results confirmed that the effect of *LINC02582* on radioresistance was at least partly dependent on USP7 and CHK1.

### MiR-200c enhances radiosensitivity by downregulation of CHK1

**Fig. 6:**
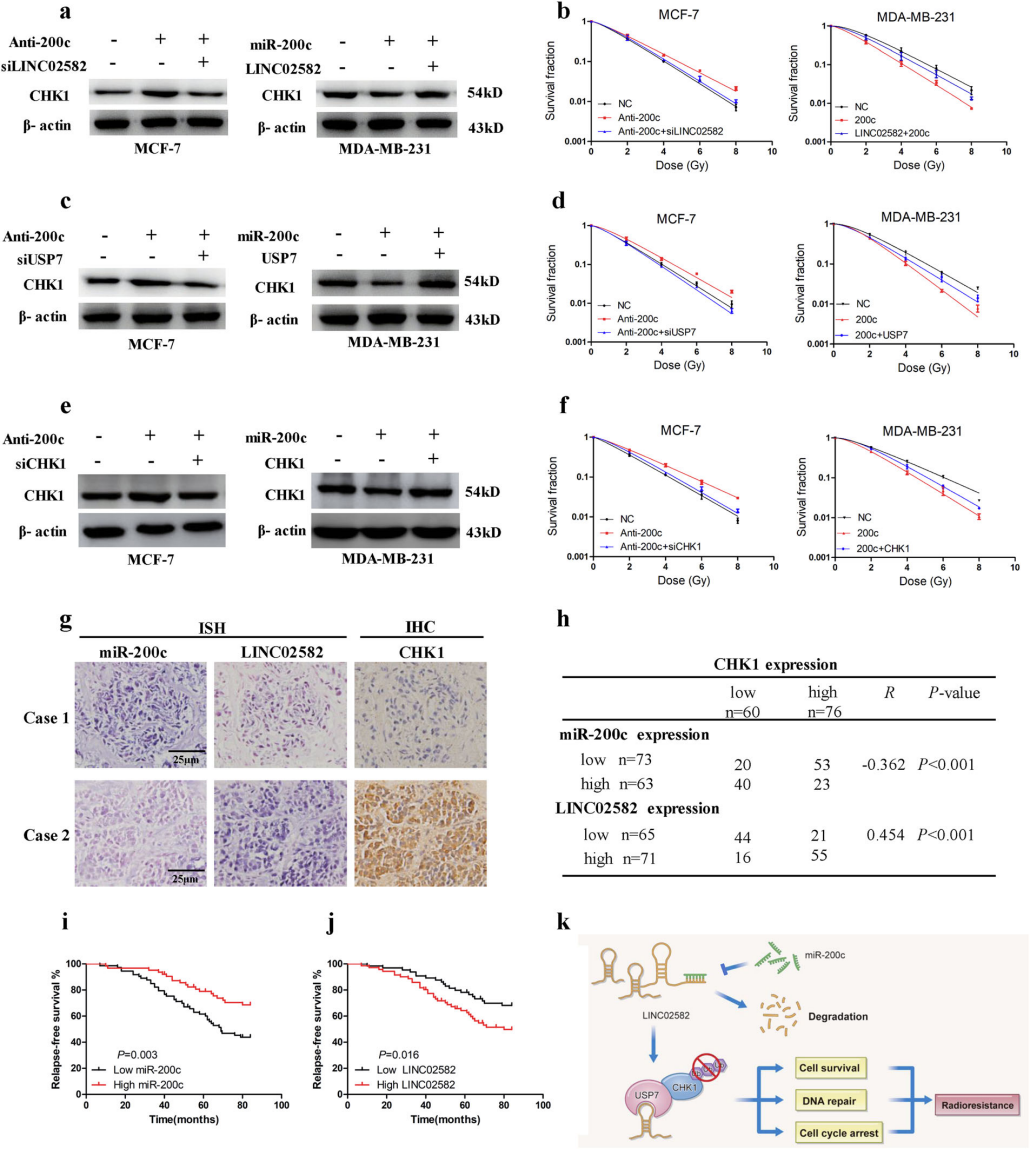
MiR-200c increases radiosensitivity through downregulation of CHK1. **a** Western blotting analysis of CHK1 protein levels. MCF-7 cells were co-transfected with an miR-200c inhibitor and *LINC02582* siRNA1; MDA-MB-231 cells were co-transfected with an miR-200c mimic and *LINC02582*. **b** The survival fraction of MDA-MB-231 and MCF-7 cells. MDA-MB-231 and MCF-7 cells treated as in **a** and then exposed to irradiation. **c** The CHK1 protein level was detected by western blotting. MCF-7 cells were co-transfected with an miR-200c inhibitor or with USP7 siRNA; MDA-MB-231 cells were co-transfected with an miR-200c mimic or with the USP7 expression plasmid. **d** The survival fraction of MDA-MB-231 and MCF-7 cells. MDA-MB-231 and MCF-7 cells treated as in **c** and then exposed to irradiation. **e** Western blotting analysis of CHK1 protein expression. MCF-7 cells were co-transfected with an miR-200c inhibitor or with CHK1 siRNA; MDA-MB-231 cells were co-transfected with an miR-200c mimic and the CHK1 expression plasmid. **f** Clonogenic survival assays of MCF-7 and MDA-MB-231 cells. MCF-7 and MDA-MB-231 cells were treated as in **e** and then exposed to irradiation. **g** MiR-200c and *LINC02582* expression were detected via in situ hybridization. CHK1 expression was detected via immunohistochemistry. **h** Correlations between the expression of miR-200c, *LINC02582*, and CHK1 in 136 clinical breast cancer specimens. The *p* values were obtained using the *χ*^2^ test. **i** Kaplan–Meier curves showing recurrence-free survival of 136 breast cancer patients who were high or low for miR-200c expression. **j** Kaplan–Meier curves showing recurrence-free survival of 136 breast cancer patients who were high or low for *LINC02582* expression. **k** Working model of the regulation of radiosensitivity by miR-200c

We sought to determine whether miR-200c could regulate radiosensitivity through CHK1. We found that suppression of miR-200c elevated CHK1 protein levels while ectopic expression of miR-200c decreased CHK1 levels. Knockdown of *LINC02582* blocked the CHK1 upregulation induced by miR-200c suppression, and *LINC02582* ectopic expression rescued the CHK1 downregulation induced by miR-200c ectopic expression (Fig. 6a). Importantly, knockdown of *LINC02582* prevented the induction of radioresistance caused by inhibition of miR-200c, while upregulation of *LINC02582* reversed the radiosensitivity caused by overexpression of miR-200c (Fig. 6b). Furthermore, knockdown of USP7 blocked the upregulation of CHK1 induced by miR-200c suppression and USP7 ectopic expression rescued the downregulation of CHK1 induced by miR-200c ectopic expression (Fig. 6c). Interestingly, knockdown of USP7 prevented the induction of radioresistance caused by inhibition of miR-200c, while upregulation of USP7 reversed the radiosensitivity caused by overexpression of miR-200c (Fig. 6d). Moreover, knockdown of CHK1 prevented the induction of radioresistance caused by inhibition of miR-200c, while upregulation of CHK1 reversed the radiosensitivity caused by overexpression of miR-200c (Fig. 6e, f). Taken together, these findings demonstrated that miR-200c enhances the radiosensitivity of breast cancer cell by downregulating the miR-200c/*LINC02582*/USP7/CHK1 signaling axis, thereby playing a critical role in regulating the radiosensitivity of breast cancer cells.

### MiR-200c and *LINC02582* expression correlate with CHK1 protein expression in breast cancer

To confirm the association between miR-200c, *LINC02582*, and CHK1 in breast cancer specimens, we performed ISH or IHC to examine the expression of these molecules in 136 breast cancer tissue samples from patients with breast cancer who had received radiotherapy (Fig. 6g). Decreased expression of miR-200c was associated with elevated CHK1 expression. However, a positive correlation between the expression of *LINC02582* and CHK1 was observed in these breast cancer specimens. Briefly, 72.6% (53/73) of the tumors with low miR-200c expression exhibited high CHK1 expression, and 77.4% (55/71) of the tumors with high *LINC02582* expression showed high CHK1 expression (Fig. 6h). Neither clinical or pathological characteristics were correlated with the expression of *LINC02582* (Supplementary Table 4). Kaplan–Meier analysis showed that patients with low miR-200c expression had significantly worse recurrence-free survival and patients with high *LINC02582* expression exhibited worse recurrence-free survival (Fig. 6i, j).

## Discussion

The results of the present study indicated that ectopic expression of miR-200c led to widespread alterations in lncRNA expression in breast cancer cells. We identified *LINC02582* as a downstream target of miR-200c. *LINC02582* is required for radioresistance in breast cancer cells. Mechanistically, *LINC02582* interacts with USP7 to deubiquitinate and stabilize CHK1, thus promoting radioresistance. These findings indicated that the miR-200c/*LINC02582*/USP7/CHK1 signaling axis play a critical role in regulating the radiosensitivity of breast cancer cells (Fig. 6k).

There is accumulating evidence that aberrant expression of lncRNAs is associated with several types of cancer, and lncRNAs play a key role in a wide range of cancer biological processes^33,34^. However, the effect of lncRNAs in radiosensitivity is largely unknown. It has been reported that miR-200c sensitizes breast cancer cells to radiation by targeting Ubiquilin 1 and TBK1 (refs. ^9,11^). However, whether miR-200c can enhance radiosensitivity through lncRNAs has not been previously investigated. In the present study, we found that overexpression of miR-200c led to widespread alteration in lncRNA expression. Among the differentially expressed lncRNAs, we identified lncRNA *LINC02582* as a downstream target of miR-200c and *LINC02582* was required for radioresistance in breast cancer cell. Breast cancer is a heterogeneous group of tumors with different estrogen receptor (ER)/progesterone receptor (PR)/human epidermal growth factor receptor 2 (HER2) statuses. In the present study, we found that knockdown of *LINC02582* in MDA-MB-231 and BT549 cells (both ER–/PR–/HER2–) significantly increased radiosensitivity. In contrast, ectopic expression of *LINC02582* in MCF-7 cell (ER+/PR+/HER2−) and BT474 cells (ER+/PR+/HER2+) contributed to raodioresistance. These results indicated that the effects of *LINC02582* on raodiosensitivity was independent of the specific subtype of breast cancer cells. Our studies revealed that lncRNAs are an important component of the miR-200c regulatory network, and highlighted the important regulatory relationships between miRNAs and lncRNAs. However, the role of other lncRNAs showing differential expression in our microarray analysis requires further investigation in a future study.

Many studies have shown that some lncRNAs function by interacting with specific proteins^35^. In this study, we performed RNA pull-down accompanied by mass spectrometric assays to identify *LINC02582*-interacting proteins. Notably, USP7 was confirmed as a specific binding protein for *LINC02582*. USP7 is a deubiquitinating enzyme that is involved in regulating the stability of CHK1. CHK1 is a critical effector kinases in the DNA damage response that facilitates DNA damage repair and promotes radioresistance of breast cancer cells^36^. Several studies reported that CUL1- and CUL4-containing E3 ubiquitin ligase complexes target CHK1 for polyubiquitylation and degradation^37^. A recent study indicated that USP7 increases CHK1 levels by direct deubiquitination, thereby promoting radioresistance in breast cancer^31^. Therefore, we reasoned that *LINC02582* might interact with USP7 to deubiquitinate CHK1, thus promoting radioresistance. Consistently, the ubiquitination of CHK1 was markedly decreased in cells overexpressing *LINC02582*; however, USP7 inhibition blocked the deubiquitination of CHK1 induced by *LINC02582* overexpression. Overexpression of *LINC02582* enhances the interaction between USP7 and CHK1. Moreover, either knockdown of USP7 or CHK1 blocked the radioresistance induced by *LINC02582* overexpression. *LINC02582* promotes radioresistance through, at least partly, USP7 and CHK1. Our results suggested that *LINC02582* enhances the interaction between USP7 and CHK1, thereby increasing the deubiquitination of CHK1 and promoting radioresistance.

MiR-200c inhibits DNA repair after radiation and acts as a radiosensitizer in breast cancer cells^9^. CHK1 facilitates DNA damage repair and promotes radioresistance^31,38^. Importantly, CHK1 is overexpressed in breast cancer^39^. CHK1 inhibition is a promising radiosensitizing strategy for breast cancer. Here, we found that expression of miR-200c reduced the CHK1 protein level, while inhibition of miR-200c upregulated the CHK1 protein level. Ectopic expression of CHK1 reversed the radiosensitivity induced by miR-200c. These results indicated that the effect of miR-200c on radiosensitivity in breast cancer cells is at least partly mediated by CHK1. We also identified *LINC02582* as a link between miR-200c, CHK1, and radioresistance of breast cancer cells. To the best of our knowledge, this is the first report to identify CHK1 as a downstream factor regulated by miR-200c. The miR-200c/*LINC02582*/USP7/CHK1 signaling axis plays important role in regulating the radiosensitivity of breast cancer cells.

We identified a negative correlation between miR-200c and CHK1 expression, as well as a positive correlation between *LINC02582* and CHK1 expression, in clinical breast cancer tissue samples. Importantly, in patients with breast cancer who had received radiotherapy, tumors showing low miR-200c or high *LINC02582* expression had much worse recurrence-free survival. However, whether miR-200c and *LINC02582* expression influences the overall survival of patients with breast cancer requires further investigation.

In summary, we demonstrated that *LINC02582* is a downstream target of miR-200c. *LINC02582* is required for radioresistance in breast cancer cells. Mechanistically, *LINC02582* interacts with USP7 to deubiquitinate and stabilize CHK1, thus promoting radioresistance. Our results suggested that the miR-200c/*LINC02582*/USP7/CHK1 signaling axis is a potential target to improve the response of breast cancer to radiation therapy.

## Material and methods

### Patient and tumor specimens

Paraffin-embedded samples of carcinomas were obtained from 136 patients with breast cancer in three hospitals, including Nanfang Hospital of Southern Medical University (Guangzhou 510515, China), Zhujiang Hospital of Southern Medical University (Guangzhou 510282, China), and Zhejiang Cancer Hospital (Hangzhou 310022, China), from January 2005 and January 2008. All these patients had received radiotherapy after surgery. Fresh tumor tissues were obtained from 42 breast cancer cases in Nanfang Hospital of Southern Medical University. All samples were collected after the patients signed informed consent according to the internal review and ethics boards of these hospitals. The diagnosis of breast cancer was confirmed histopathologically.

### Cell culture

MCF-10A cells were obtained from the American Tissue Culture Collection (ATCC, Manassas, VA, USA). MDA-MB-231, MCF-7, BT549, SKBR3, T47D, and BT474 cells were obtained from the Cell Bank of Type Culture Collection (Chinese Academy of Sciences, Shanghai, China). MCF-10A cells were cultured in Dulbecco’s modified Eagle’s medium (DMEM)/F12 (1:1) medium supplemented with 5% horse serum (Hyclone, Logan, UT, USA), 100 ng/ml cholera toxin (Sigma-Aldrich, St. Louis, MO, USA), 20 ng/ml epidermal growth factor (EGF; Peprotech, Rocky Hill, NJ, USA), 0.5 μg/ml hydrocortisone (Sigma-Aldrich), and 10 μg/ml insulin (Sigma-Aldrich). MCF-7, SKBR3 and T47D cells were maintained in DMEM containing 10% fetal bovine serum (FBS; Gibco, Melbourne, Australia). All other cell lines were cultured in Roswell Park Memorial Institute (RPMI)-1640 medium containing 10% FBS. All the cells were maintained at 37 °C in a humidified incubator containing 5% CO_2_.

### Microarrays and computational analysis

MiR-200c-overexpressing MDA-MB-231 cells and control group MDA-MB-231 cells were selected for microarray analysis. Briefly, total cellular RNA was extracted using the TRIzol Reagent (Invitrogen, Carlsbad, CA, USA) and purified using a RNeasy Mini Kit (Qiagen, Valencia, CA, USA). Then, complementary DNA was synthesized and labeled before microarray hybridization (Arraystar, Rockville, MD, USA). The slides were washed and then scanned using an Agilent DNA Microarray Scanner (Agilent, Santa Clara, CA, USA). Data were extracted using Agilent Feature Extraction software and further analyzed using Agilent GeneSpring GX, version 12.1, software. Volcano plot filtering (fold change > 2.0; *P* *<* 0.05) between miR-200c-overexpressing MDA-MB-231 cells and control MDA-MB-231 cells was performed to identify lncRNAs and mRNAs showing significantly different expression. Hierarchical clustering was performed using Cluster Treeview software (Stanford, CA, USA). The microarray data have been deposited in the National Center for Biotechnology Information (NCBI) Gene Expression Omnibus (GEO) under accession number GSE119090.

### Lentiviral construction and transduction

The miR-200c-3p lentiviral expression vectors were constructed by Genepharma (Shanghai, China). Genechem (Shanghai, China) constructed the lentiviral vectors expressing full-length human *LINC02582*. To generate clones stably overexpressing miR-200c, MDA-MB-231 and BT549 cells were infected with a lentiviral vectors expressing miR-200c-3p or an empty lentiviral vector control. To generate clones stably overexpressing *LINC02582*, MCF-7 and BT474 cells were infected with a lentiviral vector expressing *LINC02582* or an empty lentiviral vector control.

The U6-sh-*LINC02582*-CMV-GFP lentiviral vectors were constructed by Genechem (Shanghai, China) and were used to knock down the LINC02582 expression. The negative control lentiviral vector containing nonsilencing short hairpin RNA (shRNA) was used. To knock down *LINC02582*, MDA-MB-231 cells were infected with either of the lentiviral vectors encoding specific short hairpin RNA sequences or the negative control vector. Stable clones were selected for 2 weeks using puromycin (Sigma-Aldrich), and miR-200c or *LINC02582* expression were detected using quantitative real-time PCR (qRT-PCR). The shRNA sequences are described in Supplementary Table 5.

### Plasmid construction and transfection

The USP7, CHK1, and Ubiquitin expression plasmids were obtained from FulenGen (Guangzhou, China). For the transient transfection, cells were seeded on a six-well culture plate overnight and transfected with the plasmids or vector control by Lipofectamine 3000 (Invitrogen) according to the manufacturer’s instructions.

### Oligonucleotides, siRNAs transfection

The miR-200c mimic, inhibitors, USP7 small interfering RNA (siRNA), *LINC02582* siRNA, and CHK1 siRNA were purchased from RiBoBio (Guangzhou, China). Cells were seeded on the six-well culture plate and reached 50% confluence on the second day. Transfection was performed using Lipofectamine 3000 reagent (Invitrogen) according to the manufacture’s protocol. After 48 h of transfection, the cells were used for functional assays. The sequences of siRNA are listed in Supplementary Table 5.

### Quantitative real-time PCR

Total RNA was extracted from breast cancer tissues or cells using the TRIzol Reagent (Invitrogen), following the manufacturer’s instructions. qRT-PCR was performed using the SYBR Green PCR kit from Takara Biotechnology (Takara, Dalian, China). For miRNA quantification, U6 was used as an internal control. GAPDH was employed as an endogenous control for quantification of lncRNA and the mRNA levels of other genes. Primer sequences are listed in Supplementary Table 6.

### 5′ and 3′ rapid amplification of cDNA ends

Total RNA was extract from MDA-MB-231 cells as described about. Subsequently, 5′-rapid amplification of complementary DNA ends (RACE) was performed using a 5′-Full RACE Kit with TAP (Takara); 3′-RACE was performed using a 3′-Full RACE Core Set with PrimeScript RTase Kit (Takara), according to the manufacturer’s instructions.

### Luciferase assay

To construct plasmids used in dual-luciferase reporter assays, wild-type *LINC02582* cDNA (containing miR-200c-binding site) and a mutant *LINC02582* sequence (mutant in miR-200c-binding site) were cloned into pLUC-REPORT Vector (Promega, Madison, WI). Luciferase activities were assessed using a Luciferase Assay Kit (Promega), according to the manufacturer’s instructions. Briefly, MDA-MB-231 cells were co-transfected with a luciferase reporter vector (either pLUC-WT-*LINC02582* or pLUC-MUT-*LINC02582*) and mimic of miR-200c or negative control miRNA. The cells harvested and lysed for luciferase assays at 48 h after transfection. Firefly luciferase activity was used for normalization.

### Antibodies and western blotting analysis

Primary antibodies included anti-USP7, anti-Ubiquitin, anti-CHK1, anti-γ-H2AX, anti-AGO2, anti-Flag, anti-HA, anti-Myc (Cell Signaling Technology, Beverly, MA, USA), and anti-β-actin (CWBIO, Guagnzhou, China). Cell pellets were lysed with radioimmunoprecipitation assay (RIPA) buffer (Cell Signaling Technology) containing proteinase and phosphatase inhibitor cocktails (Sigma-Aldrich). The resultant proteins were electrophoresed and transferred to a nitrocellulose membrane (Bio-Rad). Membranes were blocked with 5% bovine serum albumin for 1 h before incubation with primary antibodies overnight at 4 °C, followed by incubation with horseradish peroxidase-conjugated secondary antibody and development with an ECL western blotting substrate (Pierce, Rockford, IL, USA).

### Immunofluorescence assay

Immunofluorescence was performed by counting the γ-H2AX foci at 24 h after irradiation. Breast cancer cells were seeded in 24-well culture plates and exposed to 6 Gy of irradiation. After 24 h, the cells were fixed in 4% paraformaldehyde and permeabilized in 0.1% Triton X-100 (Sigma). The cells were then blocked in 1% goat serum and incubated with primary anti-γ-H2AX antibodies (Cell Signaling Technology). Subsequently, the primary antibodies were washed off and the cells were incubated with secondary antibodies conjugated to fluorescein isothiocyanate. Finally, the cells were then incubated with 2-(4-amidinophenyl)-1H-indole-6-carboxamidine to stain the nuclei. γ-H2AX foci were visualized under a fluorescence microscope (Olympus BX51, Tokyo, Japan). The γ-H2AX foci were counted for at least 50 cells in each group.

### Clonogenic survival assay

Equal numbers of cells were seeded in six-well culture plates (pretreated with siRNAs or oligonucleotides, or plasmid transfected for 48 h) in triplicate and exposed to the indicated doses of irradiation using 6-MV X-rays from linear accelerators (Varian2300EX; Varian, Palo Alto, CA, USA) at a dose rate of 5 Gy/min. After incubation at 37 °C for 14–21 days, the plates were fixed with 100% methanol and then stained with 1% crystal violet. Colonies containing >50 cells were counted by microscopic inspection. The surviving fraction (SF) was calculated. A multitarget single-hit model was fitted to the data to generate survival curves using the following formula: SF = 1 − (1 − *e*^−*D*/*D*0^)^*N*^.

### Tumor radiosensitivity study

All the animal experiments were carried out in strict accordance with the principles and procedures approved by the Committee on the Ethics of Animal Experiments of Southern Medical University (Guangzhou, China). Suspensions of 1 × 10^6^/0.2 ml *LINC02582*-silencing or control MDA-MB-231 cells were inoculated subcutaneously into the right hindlimbs of 4-week-old female BALB/c-nu/nu nude mice. Mice were randomly assigned to no irradiation or irradiation groups (*n* = 4 mice per group). Irradiation treatment was initiated when tumors grew to approximately 150 mm³. Mice in the irradiation groups were irradiated with 2 Gy every other day for five treatments. Tumor sizes were calculated every 2 days using the formula: (length × width^2^)/2.

### RNA pull-down

RNA pull-down and deletion mapping were performed as previously described^24^. Briefly, biotinylated *LINC02582* or antisense RNA were in vitro transcribed with the Biotin RNA Labeling Mix (Roche Diagnostics, Indianapolis, IN, USA) and T7 RNA polymerase (Roche Diagnostics), treated with RNase-free DNase I (Roche Diagnostics), and purified with the RNeasy Mini Kit (Qiagen). One milligram of protein from MDA-MB-231 cell extracts was then mixed with 40 pmol of biotinylated RNA. Incubated with streptavidin agarose beads (Invitrogen) at room temperature and washed. The associated proteins were resolved by sodium dodecyl sulfate-polyacrylamide gel electrophoresis (SDS-PAGE), and then silver-stained. Specific bands were excised and analyzed using mass spectrometry.

### RNA immunoprecipitation

RIP experiments were carried out according to the manufacturer’s protocol of the Magna RIP RNA-Binding Protein Immunoprecipitation Kit (Millipore, Bedford, MA). Briefly, cells were lysed with RIPA buffer (Cell Signaling Technology) containing proteinase and phosphatase inhibitor cocktails (Sigma-Aldrich). Magnetic beads (Invitrogen) were pre-incubated with primary antibodies or anti-rabbit IgG (Cell Signaling Technology) for 30 min, and lysates were immunoprecipitated with beads, rotated overnight at 4 °C. RNA was purified from RNA–protein complexes bounded to the beads and then was analyzed by qRT-PCR.

### In vivo ubiquitination assay

For the in vivo ubiquitination assay, the 293 T cells were co-transfected with HA-tagged Ubiquitin, Flag-tagged CHK1, Myc-tagged USP7, *LINC02582* expression plasmids, and exposed to 10 µM proteasome inhibitor MG132 (Selleck, Shanghai, China) for 16 h. The cells were lysed with RIPA buffer (Cell Signaling Technology) containing proteinase and phosphatase inhibitor cocktails (Sigma-Aldrich). After centrifugation 12,000 r.p.m. for 15 min, extracts were incubated with Protein G-Plus Agarose beads (Santa Cruz Biotechnology), anti-Flag antibody (Cell Signaling Technology) was added to whole-cell lysate, and rotated overnight at 4 °C. Then, the extracts were washed with lysis buffer for four times; 40 μl of Protein G-Plus Agarose beads was added for another 2 h. The agarose beads were washed and then subjected to western blotting analysis using the indicated antibodies.

### Immunohistochemical staining

Immunohistochemistry (IHC) was performed on 4-μm-thick sections prepared using tissue blocks embedded in paraffin. The tissue sections were fixed using 4% formaldehyde overnight and then embedded in paraffin. After deparaffinization and hydration, the sections were pretreated with sodium citrate buffer in a microwave for antigen retrieval and blocked using normal goat serum. The sections were then stained using rabbit anti-CHK1 antibodies (Cell Signaling Technology) overnight at 4 °C, and then incubated in biotinylated goat anti-rabbit IgG secondary antibodies for 1 h. Finally, the sections were stained with an avidin-biotin peroxidase complex (GeneTex, Irvine, CA, USA). Two independent pathologists performed the section scoring. The score standard for the staining extent was 1 (1–25%), 2 (26–50%), 3 (51–75%) and 4 (76–100%); and 0 (negative), 1 (weak), 2 (medium) and 3 (strong) for the staining intensity. The total scores included the extent and intensity scores ranged from 1 to 7. Total scores of ≥4 defined as the high-expression group.

### In situ hybridization

In situ hybridization (ISH) was used to detect miR-200c and *LINC02582* in clinical breast cancer specimens, and was performed as previously described^11^. Briefly, the sections were deparaffinized using xylene, rehydrated in serial dilutions of ethanol, and treated with 0.2 N HCl for 5 min. The sections were incubated in proteinase K (40 µg/ml; Promega) for 20 min after washing three times. The sections were then washed in phosphate-buffered saline containing 0.2% glycine, and fixed using 4% paraformaldehyde for 10 min. The sections were reconstituted using a hybridization solution and incubated at 56 °C overnight with a digoxigenin-labeled locked nucleic acid (LNA)-miR-200c probe or digoxigenin-labeled LNA-*LINC02582* probe (Exiqon, Vedbaek, Denmark). The sections were washed twice with 5× saline-sodium citrate (SSC), buffer for 20 min each time at room temperature, followed by three washes with 2× SSC for 20 min at 50 °C. Finally, the sections were blocked with 5% normal goat serum for 1 h at room temperature before incubation with an anti-digoxigenin alkaline phosphatase conjugate (Roche, Stockholm, Sweden) overnight at 4 °C. After staining with 5-bromo-4chloro-3indolyl phosphate (BCIP)/nitro-blue tetrazolium chloride (NBT) buffer for 10 min, colorimetric signals were obtained by incubating the sections in BCIP/NBT buffer in the dark for 4 h at room temperature. Nuclear fast red was used as the counterstain. Digoxigenin-labeled LNA-scrambled miRNA or digoxigenin-labeled LNA-scrambled lncRNA probes were used as negative controls. Two independent pathologists scored the sections. The score standard for the staining extent was 1 (1–25%), 2 (26–50%), 3 (51–75%) and 4 (76–100%); and 0 (negative), 1 (weak), 2 (medium) and 3 (strong) for the staining intensity. The total scores included the extent and intensity scores ranged from 1 to 7. Total scores of ≥4 defined as the high-expression group.

### Statistical analysis

All data are expressed as the mean ± SD and statistical analysis of the data was performed using Student’s *t*-test or analysis of variance (ANOVA). Spearman rank correlation was used to analyze correlations between miR-200c and *LINC02582*. Correlation between CHK1 and miR-200c or *LINC02582* was analyzed using *χ*^2^ test. Relapse-free survival curves were plotted using the Kaplan–Meier method and compared using the log-rank test. All analyses were completed using SPSS 19.0 software (IBM Corp, Armonk, NY, USA) and *p* values < 0.05 were considered statistically significant.

## Supplementary information

Supplementary Figure 1

Supplementary Figure 2

Supplementary Figure 3

Supplementary Figure 4

Supplementary Legends

Supplementary Table 1

Supplementary Table 2

Supplementary Table 3

Supplementary Table 4

Supplementary Table 5

Supplementary Table 6
