# Supplementary figures and images for: Long noncoding RNA LINC02582 acts downstream of miR-200c to promote radioresistance through CHK1 in breast cancer cells
Source: Cell Death Dis. 2019 Oct 10;10(10):764. doi: 10.1038/s41419-019-1996-0 (PMC6787210; doi:10.1038/s41419-019-1996-0)

Supplementary Figure 1

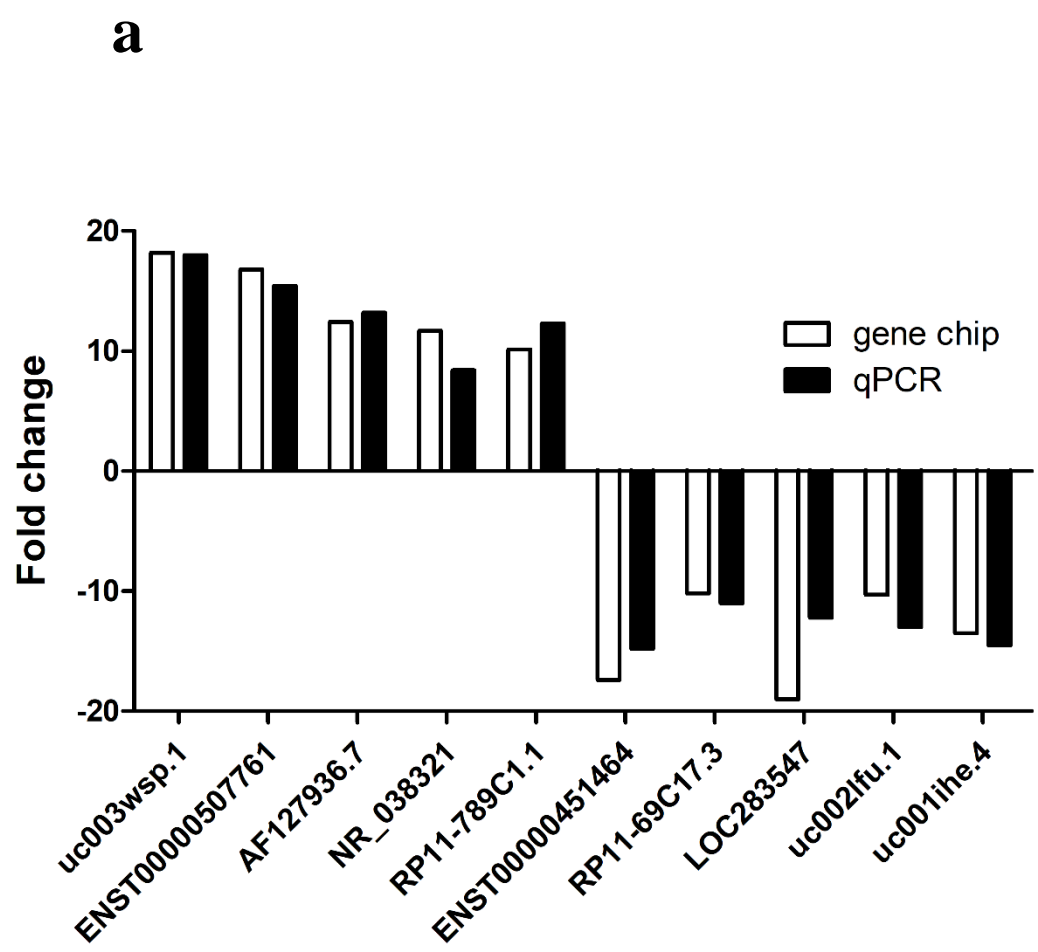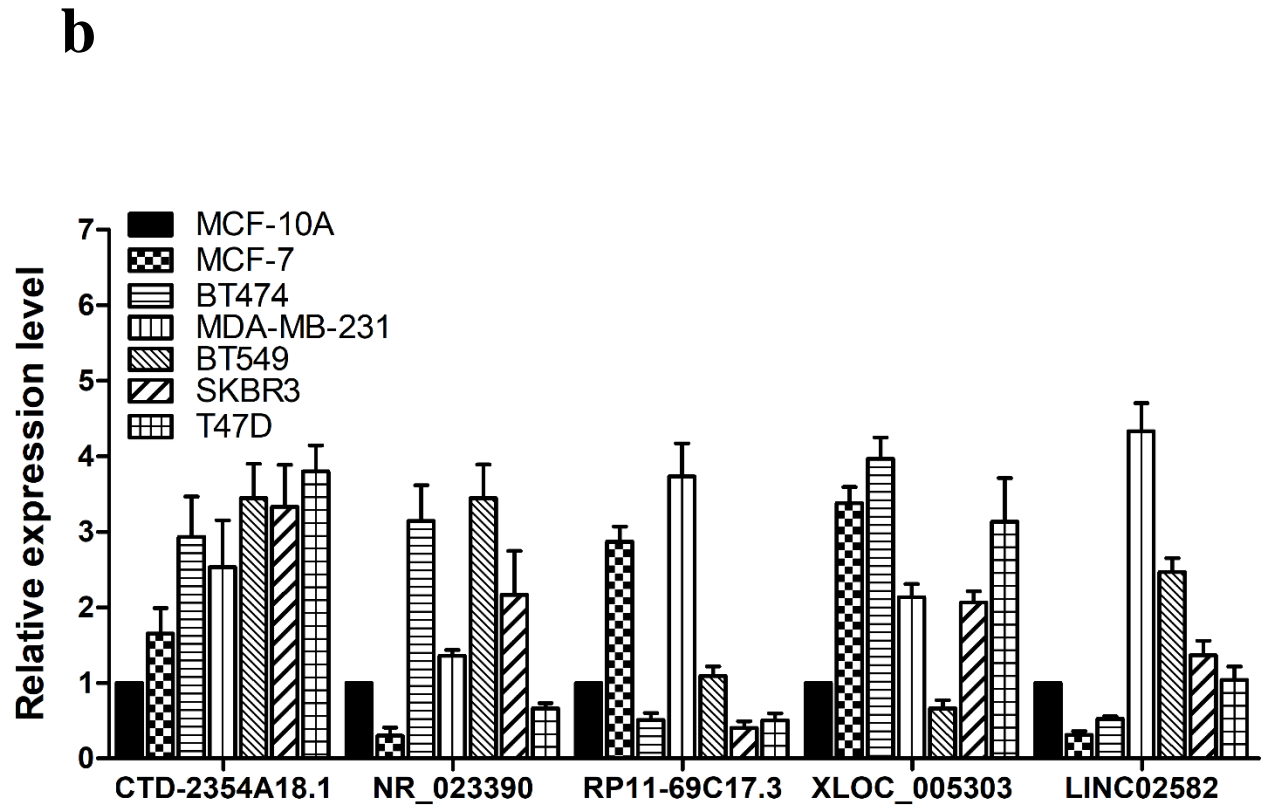

Supplement: Supplementary file 1 — Supplementary Figure 1 [file 41419_2019_1996_MOESM1_ESM.pdf]

Supplementary Figure 2

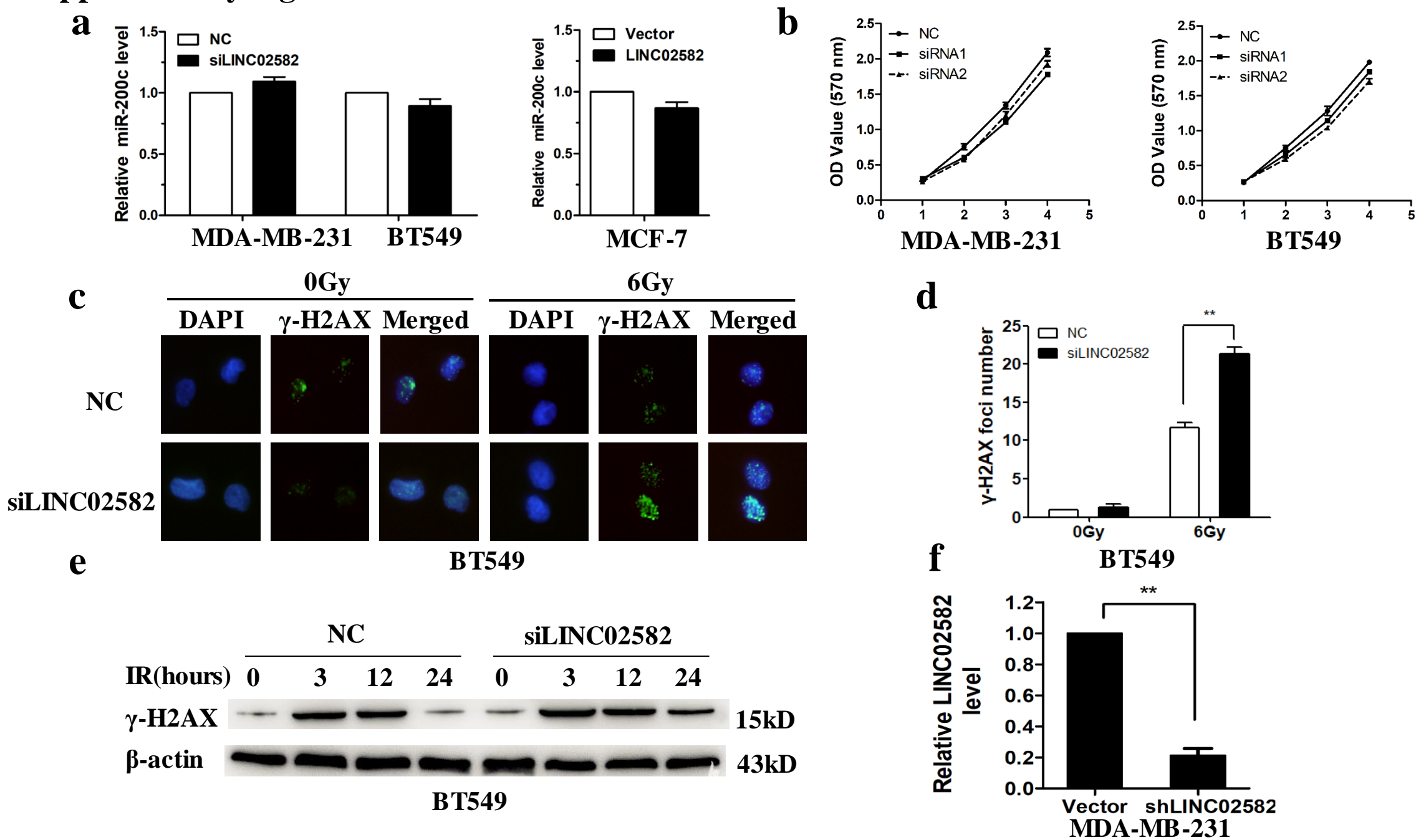

Supplement: Supplementary file 2 — Supplementary Figure 2 [file 41419_2019_1996_MOESM2_ESM.pdf]

Supplementary Figure 3

**a**

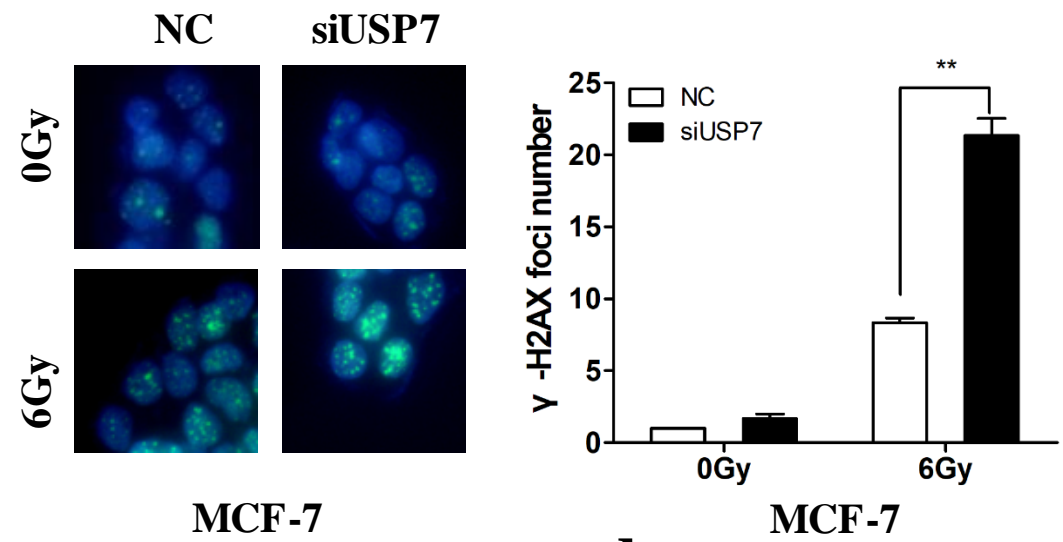

**b**

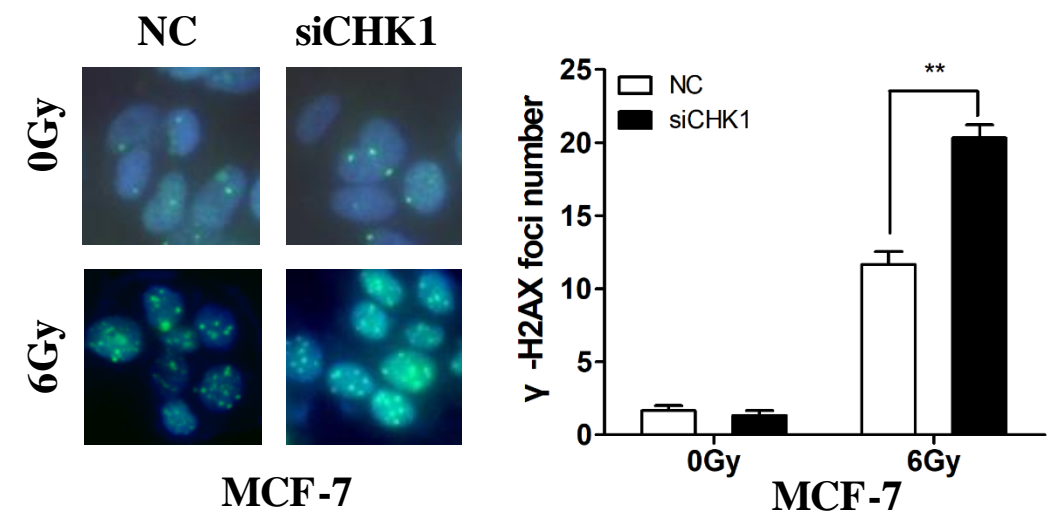

**c**

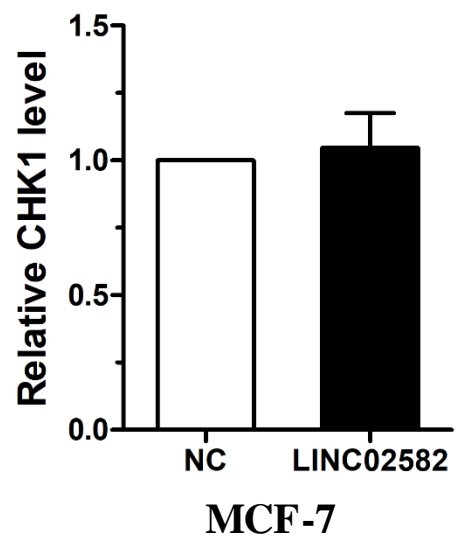

**d**

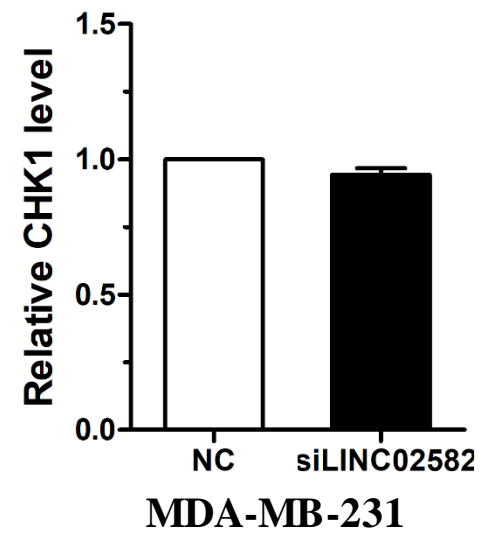

Supplement: Supplementary file 3 — Supplementary Figure 3 [file 41419_2019_1996_MOESM3_ESM.pdf]

Supplementary Figure 4

a

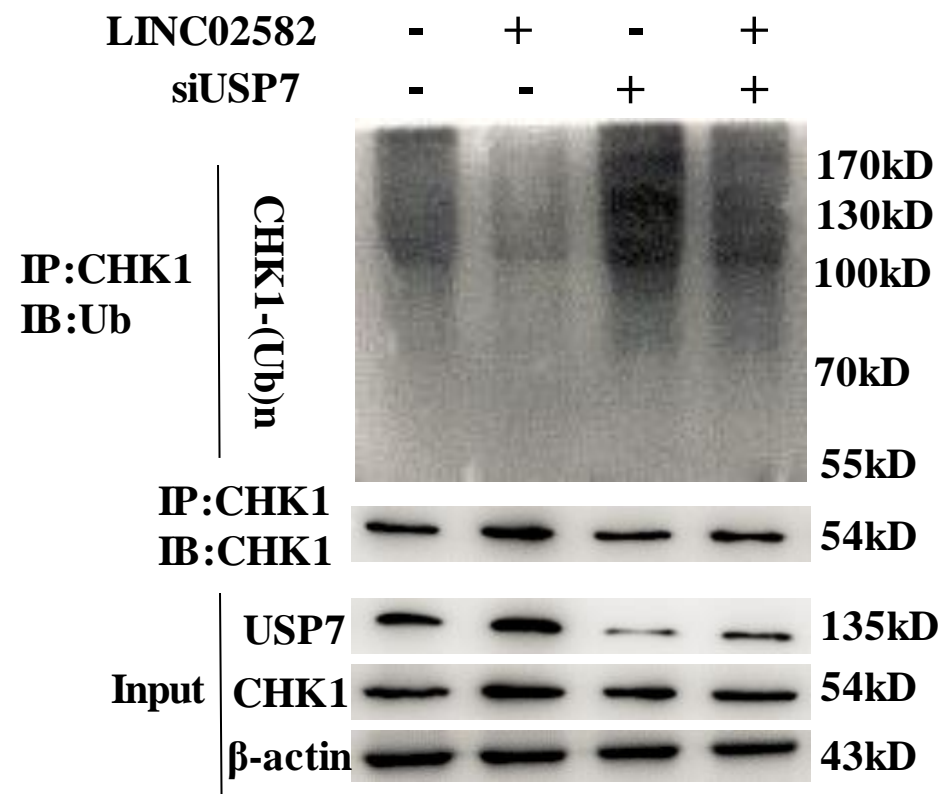

Supplement: Supplementary file 4 — Supplementary Figure 4 [file 41419_2019_1996_MOESM4_ESM.pdf]
