# Supplementary material for: Long noncoding RNA LINC02582 acts downstream of miR-200c to promote radioresistance through CHK1 in breast cancer cells
Source: Cell Death Dis. 2019 Oct 10;10(10):764. doi: 10.1038/s41419-019-1996-0 (PMC6787210; doi:10.1038/s41419-019-1996-0)
Supplement: Supplementary file 5 — Supplementary Legends [file 41419_2019_1996_MOESM5_ESM.pdf]

Supplementary Figure Legends

Figure 1. Induction of miR-200c leads to widespread alteration in lncRNA expression.

(a) Differential expression of 10 lncRNAs in miR-200c-overexpressing cells and control cells validated by qRT-PCR. (b) Relative expression of candidate lncRNAs in breast cancer cells and MCF-10A cells.

Figure 2. *LINC02582* contributes to radioresistance of breast cancer cells.

(a) Left, miR-200c expression levels in MDA-MB-231 and BT549 cells after inhibited *LINC02582*. Right, miR-200c expression levels in MCF-7 cells after *LINC02582* overexpression. (b) MDA-MB-231 and BT549 cells were transfected with two different *LINC02582* siRNAs, cell growth was determined at day 1-4 after transfection. (c-d) Formation of γ-H2AX foci at 24 h after 6 Gy IR, analyzed by immunofluorescence in BT549 cells transfected with *LINC02582* siRNA1. (e) Western blotting analysis of γ-H2AX expression in BT549 cells transfected with *LINC02582* siRNA1, at the indicated time points after 6 Gy IR. (f) Relative expression of *LINC02582* in MDA-MB-231 cells after transduction of lentiviruses encoding *LINC02582* short hairpin RNA (shRNA). Data are presented as means ± SD, n=3, *P <0.05, **P <0.01.

Figure 3. MiR-200c regulates *LINC02582* expression in breast cancer cells.

(a) γ-H2AX foci formation analyzed using the immunofluorescence assay in MCF-7 cells transfected with USP7 siRNA, 24 h after 6Gy IR. (b) γ-H2AX foci formation analyzed using the immunofluorescence assay in MCF-7 cells transfected with CHK1 siRNA, 24 h after 6Gy

IR. (c) Relative expression of CHK1 mRNA in MCF7 cells after *LINC02582* overexpression. (d) Relative expression of CHK1 mRNA in MDA-MB-231 cells after inhibited *LINC02582*. Data are presented as means ± SD, n=3, * *P* <0.05, ***P* <0.01.

Figure 4. *LINC02582* promotes CHK1 deubiquitination through USP7.

(a) MCF-7 cells erexpressing *LINC02582* were transfected with USP7 siRNA. After transfection, the cells were treated with MG132 (10 μM) for 16 h. Lysates were immunoprecipitated with anti-CHK1 antibody and the immunoprecipitates and input were analyzed by western blotting with the indicated antibodies.
