# Supplementary material for: Long noncoding RNA LINC02582 acts downstream of miR-200c to promote radioresistance through CHK1 in breast cancer cells
Source: Cell Death Dis. 2019 Oct 10;10(10):764. doi: 10.1038/s41419-019-1996-0 (PMC6787210; doi:10.1038/s41419-019-1996-0)
Supplement: Supplementary file 6 — Supplementary Table 1 [file 41419_2019_1996_MOESM6_ESM.pdf]

Supplementary Table 1. Target lncRNAs for has-miR-200c-3p

	Target			Sites	TargetScan		miRanda			Annotations				
**Seqname**	**GeneSymbol**	**Fold chang**	**Regulation**	**Total**	**Context+**	**Context**	**Structure**	**Energy**	**Site_type**	**site_start**	**site_end**	**TR Leng**	**chrom**	**strand**
ENST0000058394	CTD-2354A18.	30.38	down	1	-0.138	-0.249	142	-11.37	7mer-m8	616	638	847	chr18	+
NR_038340	LINC02582	19.78	down	1	-0.106	-0.229	142	-11.37	7mer-m8	419	441	1181	chr18	+
ENST0000042107	RP11-69C17.3	9.39	down	1	-0.212	-0.389	164	-19.58	7mer-m8	381	405	2321	chr10	+
NR_023390	LINC00476	9.22	down	1	-0.087	-0.186	155	-18.33	7mer-m8	7862	7883	8030	chr9	-
TCONS_0001182	XLOC_005303	7.77	down	1	-0.161	-0.296	150	-13.75	7mer-m8	290	315	597	chr6	+
ENST0000056510	RP11-65L3.1	6.13	down	1	-0.014	0.003	147	-19.37	7mer-m8	41	61	2058	chr2	+
ENST0000053170	C6orf3	4.62	down	1	-0.057	-0.159	140	-8.82	7mer-m8	344	366	2421	chr6	+
ENST0000041983	RP11-332K15.1	4.53	down	1	-0.222	-0.415	140	-10.59	8mer-1a	18	40	451	chr7	+
ENST0000047465	RP4-545C24.1	4.48	down	1	-0.011	-0.05	143	-14.77	7mer-m8	571	592	773	chr7	+
ENST0000045080	RP11-27I1.2	4.24	down	1	-0.054	-0.149	145	-12.91	7mer-m8	228	250	912	chr9	+
ENST0000056369	CTD-3105H18.	4.15	down	2	-0.019	-0.07	281	-36.39	7mer-1a	809	835	1980	chr19	-
ENST0000056150	RP11-1024P17.	4.10	down	1	-0.128	-0.238	147	-12.49	7mer-m8	101	121	1916	chr3	+
ENST0000027044	ZNF702P	4.04	down	2	-0.282	-0.568	299	-26.11	7mer-m8	2633	2655	2990	chr19	-
ENST0000045694	RP11-82L18.2	3.73	down	1	-0.191	-0.344	153	-17.02	7mer-m8	105	127	583	chr9	-
uc003zmh.1	BX648501	3.67	down	1	-0.094	-0.249	150	-10.54	7mer-m8	700	720	2421	chr9	+
ENST0000043264	RP11-27I1.2	3.66	down	1	-0.046	-0.144	145	-12.91	7mer-m8	284	306	719	chr9	+
NR_024539	POPDC3	3.58	down	1	-0.082	-0.214	162	-17.54	7mer-m8	400	420	1389	chr6	-
TCONS_0000374	XLOC_001546	3.52	down	1	-0.072	-0.165	149	-14.86	7mer-m8	272	294	464	chr2	+
uc003kkc.2	FLJ42709	3.46	down	1	-0.109	-0.287	153	-8.98	7mer-m8	1710	1730	2562	chr5	-
NR_046269	SSBP1	3.27	down	1	-0.015	-0.103	144	-9.33	7mer-m8	426	453	764	chr7	+
ENST0000056892	RP11-16P6.1	3.27	down	1	-0.013	-0.073	140	-9.6	7mer-1a	185	208	2815	chr2	-
ENST0000050749	RP11-94H18.1	3.16	down	1	-0.179	-0.301	149	-10.49	7mer-m8	1811	1832	1867	chr8	-
ENST0000055425	RP11-618G20.2	3.02	down	1	-0.072	-0.168	152	-17.27	7mer-m8	326	386	533	chr14	-
ENST0000054789	RP11-328C8.5	3.02	down	1	-0.2	-0.378	149	-15.49	8mer-1a	198	218	447	chr12	-
uc001vmm.3	AK094990	2.94	down	2	-0.073	-0.396	288	-26.8	7mer-m8	2133	2157	3106	chr13	-
NR_028131	CFL2	2.90	down	1	-0.082	-0.362	144	-11.86	8mer-1a	897	919	3013	chr14	-
TCONS_0000596	XLOC_002602	2.85	down	1	-0.056	-0.159	140	-10.59	7mer-m8	592	614	997	chr3	+
NR_028130	CFL2	2.84	down	1	-0.054	-0.343	144	-11.86	8mer-1a	1008	1030	3124	chr14	-
ENST0000056903	RP11-15N24.4	2.83	down	1	-0.071	-0.165	147	-12.82	7mer-m8	2019	2039	2164	chr3	+
NR_033810	CYP3A5	2.82	down	1	-0.03	-0.212	148	-12.67	7mer-m8	1623	1646	2859	chr7	-
ENST0000056900	RP11-434B12.1	2.75	down	2	-0.464	-0.878	301	-31.3	7mer-m8	598	621	1553	chr2	-
ENST0000048442	PLGLA	2.74	down	1	-0.048	-0.141	145	-10.44	7mer-m8	441	463	700	chr2	+
TCONS_0000981	XLOC_005086	2.71	down	1	-0.085	-0.168	140	-14.85	7mer-m8	46	68	1008	chr5	-

	Target			Sites	TargetScan		miRanda			Annotations				
**Seqname**	**GeneSymbol**	**Fold chang**	**Regulation**	**Total**	**Context+**	**Context**	**Structure**	**Energy**	**Site_type**	**site_start**	**site_end**	**TR Leng**	**chrom**	**strand**
ENST0000044789	RP11-27I1.2	2.64	down	2	-0.062	-0.282	302	-25.73	7mer-m8	429	451	3519	chr9	+
NR_038970	LINC00641	2.63	down	1	-0.157	-0.289	143	-12.13	7mer-m8	5123	5144	5439	chr8	-
NR_027942	COX11	2.58	down	2	-0.077	-0.282	298	-31.99	7mer-m8	669	692	1124	chr17	-
NR_038971	LINC00641	2.57	down	1	-0.157	-0.289	143	-12.13	7mer-m8	1843	1864	2159	chr14	-
uc003kkf.2	FLJ42709	2.54	down	1	-0.109	-0.287	153	-8.98	7mer-m8	1940	1960	2792	chr5	-
NR_027941	COX11	2.52	down	2	-0.077	-0.283	298	-31.99	7mer-m8	807	829	1138	chr17	-
uc001aze.3	CROCCP2	2.48	down	1	-0.125	-0.27	159	-17.61	7mer-m8	1754	1778	2175	chr1	-
ENST0000044499	MCM3AP-AS1	2.46	down	1	-0.007	-0.15	153	-11.93	7mer-m8	1323	1344	2193	chr21	+
ENST0000043212	RP11-119K6.6	2.42	down	1	-0.125	-0.244	155	-13.31	7mer-m8	73	94	645	chr10	+
ENST0000045205	AC007038.7	2.39	down	1	-0.018	-0.137	140	-11.01	7mer-m8	1502	1524	2199	chr2	+
ENST0000057137	RP11-199F11.2	2.38	down	1	-0.174	-0.293	148	-10.96	7mer-m8	985	1008	1112	chr17	-
ENST0000053038	CTD-3012A18.	2.31	down	1	-0.079	-0.177	156	-17.56	7mer-m8	39	59	563	chr11	+
uc002the.2	LOC541471	2.31	down	1	-0.112	-0.269	158	-13.68	7mer-m8	978	999	1583	chr2	-
TCONS_0002654	XLOC_012861	2.29	down	1	-0.08	-0.172	144	-12.74	7mer-m8	62	87	436	chr18	-
ENST0000046461	SNHG12	2.25	down	1	-0.036	-0.129	145	-10.71	7mer-m8	248	270	719	chr1	-
NR_036553	FASTKD3	2.24	down	1	-0.272	-0.465	145	-15.17	8mer-1a	645	669	900	chr5	-
uc001uxg.3	AK021977	2.22	down	1	-0.049	-0.157	145	-9.97	7mer-m8	396	418	7545	chr13	-
NR_028138	LOC338758	2.19	down	1	-0.152	-0.266	143	-11.54	7mer-m8	2875	2897	2998	chr12	+
TCONS_0001986	XLOC_009079	2.16	down	1	-0.049	-0.137	141	-16.18	7mer-m8	30	52	788	chr11	+
ENST0000050315	CTD-2201E18.	2.15	down	1	-0.033	-0.127	154	-22.94	7mer-m8	164	187	608	chr5	-
uc011cue.1	FLJ42709	2.12	down	1	-0.109	-0.287	153	-8.98	7mer-m8	1965	1985	2817	chr5	-
NR_036434	RSRC2	2.07	down	1	-0.154	-0.275	141	-15.03	7mer-m8	2237	2259	2430	chr12	-
uc003kkg.2	FLJ42709	2.06	down	1	-0.109	-0.287	153	-8.98	7mer-m8	1780	1800	2632	chr5	-
ENST0000056533	RP4-561L24.3	2.03	down	1	-0.079	-0.178	151	-13.78	7mer-m8	1573	1594	1766	chr1	+
ENST0000056841	AC137934.1	2.01	down	1	-0.001	-0.11	140	-12.44	7mer-m8	1420	1442	2056	chr16	+
