# Supplementary material for: Long noncoding RNA LINC02582 acts downstream of miR-200c to promote radioresistance through CHK1 in breast cancer cells
Source: Cell Death Dis. 2019 Oct 10;10(10):764. doi: 10.1038/s41419-019-1996-0 (PMC6787210; doi:10.1038/s41419-019-1996-0)
Supplement: Supplementary file 7 — Supplementary Table 2 [file 41419_2019_1996_MOESM7_ESM.pdf]

Supplementary Table 2. Analysis results of *LINC02582* sequences by Open Reading Frame Finder Finder from NCBI

1、Sequences of *LINC02582*

GCTGACTCTCTTTTCGGACTCAGCCCGCCTGCACCCAGGTGAAATAAACTGCCATGTTGCT
CACACTAAGCCTGTTTGGTGGTCTCTTCACAGGGACACGGATGAAATTTCGTGCCGTGACT
CGGATCGGAGAACCTCCCTTAGGAGATCAATCCCCTGTACTCCTTTTCTTTACCCTGTGAG
AAAGATCCACCTATTACCTCAGGTCCTCAGACCGACCAGCCCAAGGAACATCTCACCAATT
TTAAATGAGACCTTGAAGATTTGTTGTTCAAGGAGAAACTGAAGACCAAGAAGGAAAGTC
AGAGCCAGCAATACCAGCAGAGCCAGATCTGAGCTGGGAGAAGCCGAGAAAGTTTGTGA
ACAGGAGATCGGTGACCTGGGCTCCTTATGTGCCTGAAAGAGTTTGAGTTTCCTGTTAACT
CCAAATCAACAGTATTTTCAACAAGAAATGTGCAATTGAAATCAAGTGCTGTTTAAGTACA
GCTTGGATTTCCCCAGGAAGACACTTGCAGTGAACAGAGTTATGGAGCAGCAAAAACACA
GATCTATTTGGAATTAGAGATAAGATATGCGTTGTATTTTGCGTCAATTATATAATACCATCCT
CTCAAAGGTGGTTCTAAATTACAAAGGACTTTGATTTCTAGGTAGATTCTGGGTAGAGACT
TCCTTTCTTATTGAGGCATTAATGACACCGTTTAACCTGTGACGCAATATGACTGGAGTTGT
ACTTTGAGAAGATTAATCAGGTTTGGTTGCAGAATGAAAGAGAAGATGAAGTCAAGAGAT
TGGTTTAGAGGCTCTAGCAGAAGCTTAGTCATATTTCAAAATGATCAAATGTCAAGAAATAT
TCTGAGCTGCATAACTTGTATAAAGTAATTTTCAGTGATTTCTTTCATGGTTATGATAAAAGA
ACTGGATTCGCAGAAACTTTTACCCTGAATCAAGATTTAATTTTTCTTTGAGCTCATCTTAA
GGATATCGGAACATAGGGAGCAAACGATGGTGTGGCTGCCTCAGTGCTTGATTTTTAACGG
TTTTGAAGAGATTAGTTACATTTCTTCTCCTAGTAAGAACTAATAAATACTTTAACAGAAAT
GAATTCCCTATTTCTTTGTACACTGGTCTATGTCTTCAAAACATTAAATACTATTGATAAGAT
ATCACCTTTTAAAAAA

2、The Open Reading Frame of *LINC02582*

Mark subset... Marked: 0 Download marked set as Feature table

Label	Strand	Frame	Start	Stop	Length (nt | aa)
**ORF1**	**+**	**1**	**196**	**336**	**141 | 46**
ORF2	+	1	391	480	90 | 29
ORF3	+	1	766	882	117 | 38
ORF4	+	3	54	182	129 | 42
ORF5	+	3	1101	>1181	81 | 26
ORF6	-	1	824	702	123 | 40
ORF7	-	1	689	549	141 | 46
ORF8	-	2	571	419	153 | 50
ORF9	-	3	1092	976	117 | 38

3、The predicted protein results of *LINC02582* Open Reading Frame

>lcl|ORF1:196:336 unnamed protein product
MTSGPQTDQPKEHLTNFKSDLEDLLFKEKLKSKKESESQQYQQSQI
>lcl|ORF2:391:480 unnamed protein product
MCLKEFEFPVNSKSTVFSTRNVQLKSSAV
>lcl|ORF3:766:882 unnamed protein product
MKEKMKSRDWFRGSSRSLVIFQNDQISRKILSCITCIK
>lcl|ORF4:54:182 unnamed protein product
MLLTQSLFGGLFTGTRMKFGAVTRIGEPPLGDQSPVLLFFAL
>lcl|ORF5:1101:1181 unnamed protein product, partial
MNSLSLCTLVYFFKTLNTIDKISPFKK
>lcl|ORF6:824:702 unnamed protein product
MTKLLLEPLNQSLDFIFSFILQPNLINLLKVQLQSYCFPG
>lcl|ORF7:689:549 unnamed protein product
MPQYERKSLPRIYLEIKVLCNLEPPLRGWYFIIEAKYNAYVFSFSK
>lcl|ORF8:571:419 unnamed protein product
MFSLFPNRSVFLLLHNSVHCKCLPVEILAALKQHLISIAHFLLKILLIWS
>lcl|ORF9:1092:976 unnamed protein product
MYLLVLTRRRNVTILFKTVKNQALRQPHHRLLPMFRYP
