# Supplementary material for: Long noncoding RNA LINC02582 acts downstream of miR-200c to promote radioresistance through CHK1 in breast cancer cells
Source: Cell Death Dis. 2019 Oct 10;10(10):764. doi: 10.1038/s41419-019-1996-0 (PMC6787210; doi:10.1038/s41419-019-1996-0)
Supplement: Supplementary file 8 — Supplementary Table 3 [file 41419_2019_1996_MOESM8_ESM.pdf]

Supplementary Table 3. Mass spectrometry Analysis of the Proteins Pull Down by *LINC02582*

Rank	Name and Official Symbol	Scores	Protein Mass	No of Peptide	Relative Abundance
1	Ubiquitin specific peptidase 7 OS=Homo sapiens	647	174622	22	40.19%
2	Alpha-2-macroglobulin OS=Homo sapiens	334	164613	22	21.42%
3	Serum albumin OS=Homo sapiens	252	164613	19	20.62%
4	Hemopexin OS=Homo sapiens	74	52385	10	13.01%
5	Hemoglobin subunit beta OS=Homo sapiens	51	16102	5	8.21%
6	Actin, cytoplasmic 1 OS=Homo sapiens	36	42052	5	7.96%
7	MAP kinase-activated protein kinase 3 OS=Homo sapiens	31	37822	5	7.2%
8	Ig lambda-6 chain C region OS=Homo sapiens	19	11441	2	5.23%
9	Apolipoprotein E OS=Homo sapiens	18	30733	2	4.24%
10	Uromodulin OS=Homo sapiens	18	7350	1	3.45%
