# Supplementary material for: Long noncoding RNA LINC02582 acts downstream of miR-200c to promote radioresistance through CHK1 in breast cancer cells
Source: Cell Death Dis. 2019 Oct 10;10(10):764. doi: 10.1038/s41419-019-1996-0 (PMC6787210; doi:10.1038/s41419-019-1996-0)
Supplement: Supplementary file 9 — Supplementary Table 4 [file 41419_2019_1996_MOESM9_ESM.pdf]

Supplementary Table 4. Correlation between miR-200c, *LINC02582* and CHK1 expression level and distribution of clinicopathological factors in breast cancer patients.

	No. of patients	miR-200c expression		*P* value	*LINC02582* expression		*P* value	CHK1 expression		*P* value
		low (n=73)	high (n=63)		low (n=65)	high (n=71)		low (n=60)	high (n=76)	
Age(y)										
Mean± SD	136	48.41±11.80	51.84±10.04	0.07[a]	49.54±12.01	48.03±10.13	0.428[a]	47.67±13.22	50.26±8.98	0.177[a]
Tumor size(cm)										
<5	88	53	35	0.430 [b]	39	49	0.60 [b]	33	55	0.527 [b]
≥5	48	20	28		26	22		27	21	
Tumor grade										
G1-G2	63	30	33	0.2284[b]	28	35	0.495[b]	35	28	0.016[b]
G3	73	43	30		37	36		25	48	
Lymph node										
Negative	66	24	42	0.024[b]	34	32	0.490[b]	31	35	0.101[b]
Positive	70	49	21		31	39		29	41	

In situ hybridaization was used to detect the expression status of miR-200c and *LINC02582* (low or high).

Immunohistochemistry was used to detect the expression status of CHK1 (low or high).

[a]Compared using Student t test;

[b]Compared using χ^2 test.
