# Supplementary material for: Long noncoding RNA LINC02582 acts downstream of miR-200c to promote radioresistance through CHK1 in breast cancer cells
Source: Cell Death Dis. 2019 Oct 10;10(10):764. doi: 10.1038/s41419-019-1996-0 (PMC6787210; doi:10.1038/s41419-019-1996-0)
Supplement: Supplementary file 10 — Supplementary Table 5 [file 41419_2019_1996_MOESM10_ESM.pdf]

Supplementary Table 5. SiRNA and shRNA sequence used in this study

Genes	Sequence
LINC02582 siRNA1	5'-GAAAUCAAGUGCUGUUUAAdTdT-3'
LINC02582 siRNA2	5'-GCAGAAGCUUAGUCAUAUUdTdT-3'
LINC02582 siRNA3	5'-CAAUAUGACUGGAGUUGUAdTdT-3'
USP7 siRNA	5'-GGCAACCUUUCAGUUCACUdTdT-3'
CHK1 siRNA	5'-GGAGAGAAGGCAAUAUCCAdTdT-3'
miR-200c inhibitor	5'-UCCAUCAUUACCCGGCAGUAUUA-3'
LINC02582 shRNA	5'-GAAAUCAAGUGCUGUUUAA-3'
