# Supplementary material for: Long noncoding RNA LINC02582 acts downstream of miR-200c to promote radioresistance through CHK1 in breast cancer cells
Source: Cell Death Dis. 2019 Oct 10;10(10):764. doi: 10.1038/s41419-019-1996-0 (PMC6787210; doi:10.1038/s41419-019-1996-0)
Supplement: Supplementary file 11 — Supplementary Table 6 [file 41419_2019_1996_MOESM11_ESM.pdf]

Supplementary Table 6. Primer Sequence used in this study

Gene	Sequence (5'-3')
miR-200c-F	ACACTCCAGCTGGGCGTCTTACCCAGCAGT
miR-200c-R	TGGTGTCGTGGAGTCG
miR-200c-RT	CTCAACTGGTGTCGTGGAGTCGGCAATTCAGTTGAGCCAAAC
U6-F	CTCGCTTCGGCAGCACA
U6-R	AACGCTTCACGAATTTGCGT
U6-RT	CGCTTCACGAATTTGCGTGTCAT
LINC02582-F	AGCCCAAGGAACATCTCACC
LINC02582-R	CACCGATCTCCTCTTCACAAAC
GAPDH-F	CGGAGTCAACGGATTTGGTCGTAT
GAPDH-R	AGCCTTCTCCATGGTGGTGAAGAC
CHK1-F	GGTGAATATAGTGCTGCTATGTTGACA
CHK1-R	TTGGATAAACAGGGAAGTGAACAC
USP7-F	CATCTCTGTCACCTACTCTG
USP7-R	ACCTCCTCCTCTCATTCTC
CTD-2354A18-F	TGGGACCATTCTTTTTGCAT
CTD-2354A18-R	GTCCCTCAGGAGGTGACAAA
RP11-69C17-F	GGGGGCGGATAATATCACTT
RP11-69C17-R	CAGGCATAAGGCAGAACACA
NR_023390-F	CATCTCTGTCACCTACTCTG
NR_023390-R	ACCTCCTCCTCTCATTCTC
XLOC_005303-F	CTGATGGTGATGATGATGTC
XLOC_005303-R	TCCTGAGGTCTTGATATTCC
